# Species and genetic diversity relationships in benthic macroinvertebrate communities along a salinity gradient

**DOI:** 10.1186/s12862-022-02087-6

**Published:** 2022-11-02

**Authors:** H. Cecilie Petersen, Benni W. Hansen, K. Emily Knott, Gary T. Banta

**Affiliations:** 1 Department of Science and Environment, Roskilde University, Universitetsvej 1, 4000 Roskilde, Denmark; 2 Department of Biological and Environmental Science, University of Jyväskylä, 40014 Jyväskylä, Finland; 3 Department of Biology, University of Southern Denmark, 5238 Odense M, Denmark

**Keywords:** SGDC, Ultra-conserved elements, Species diversity, Genetic diversity, Macrobenthos, Salinity, Spatial and temporal patterns

## Abstract

**Background:**

Species- and genetic diversity can change in parallel, resulting in a species-genetic diversity correlation (SGDC) and raising the question if the same drivers influence both biological levels of diversity. The SGDC can be either positive or negative, depending on whether the species diversity and the genetic diversity of the measured species respond in the same or opposite way to drivers. Using a traditional species diversity approach together with ultra-conserved elements and high throughput sequencing, we evaluated the SGDCs in benthic macrofauna communities in the Baltic Sea, a geologically young brackish water sea characterised by its steep salinity gradient and low species richness. Assessing SGDCs from six focal marine invertebrate species from different taxonomic groups and with differing life histories and ecological functions on both a spatial and temporal scale gives a more comprehensive insight into the community dynamics of this young ecosystem and the extrinsic factors that might drive the SGDCs.

**Results:**

No significant correlations between species diversity and genetic diversity were found for any of the focal species. However, both negative and positive trends of SGDCs for the individual focal species were observed. When examining the environmental drivers, no common trends between the species were found, even when restricting the analysis to specific taxonomic classes. Additionally, there were no common environmental factors driving the diversity relationships for species sharing the same SGDC trend (positive or negative). Local population dynamics, together with the invasion history of the individual species and their unique adaptation to the distinctive environment of the Baltic Sea, are expected to be of major influence on the outcome of the SGDCs.

**Conclusions:**

The present results highlight the importance of assessing SGDCs using multiple species, not just a single indicator species. This emphasises a need to pay attention to the ecology and life history of the focal species. This study also provides insight into the large differences in both patterns and drivers of genetic diversity, which is important when including genetic biodiversity in conservation plans. We conclude that the effects of environmental and biological factors and processes that affects diversity patterns at both the community and genetic levels are likely species dependent, even in an environment such as the Baltic Sea with strong environmental gradients.

**Supplementary Information:**

The online version contains supplementary material available at 10.1186/s12862-022-02087-6.

## Background

Quantification of species diversity is a central and essential tool for describing communities and assessing biodiversity and its conservation. Genetic diversity of species is an additional component of biodiversity that provides an insight into the diversity within species and is an important element for understanding community dynamics [1]. Genetic diversity, including epigenetics, is important because it underlies the traits of organisms and allows for assessing contemporary evolution [2]. It can even reveal cryptic and pseudo-cryptic species, in otherwise morphologically undistinguishable specimens of what is believed to be the same species [3]. Species diversity and genetic diversity are likely not independent of each other, and both neutral and selective processes can influence both species and genetic diversity [4]. If the drivers of the diversity at the two biological levels are related, diversity may co-vary in a way that leads to a species-genetic diversity correlation (SGDC) [4]. Positive correlations are expected if the driving factors affect the species in which genetic diversity is measured (the focal species) and the community in a similar way. If the focal species and the community respond differently to the same driver, there can be a negative correlation or no correlation between the species and genetic diversity.

In theoretical models, SGDCs are commonly expected to be positive [4]. In a diverse range of ecosystems and species such as terrestrial plants, invertebrates, and vertebrates, both positive SGDCs [5–7], negative SGDCs [8, 9], and non-significant SGDCs [10, 11] were revealed. Meta-studies have shown positive SGDCs to be dominant in the literature supporting the theoretical assumptions [12, 13], and a recent compilation [14] found that 80% of the published SGDCs had positive correlations, although the study also suggests that this is in part due to a publication bias towards positive SGDCs.

SGDCs may be driven by extrinsic factors exterior to the community, or by intrinsic forces from within the community [14]. Extrinsic factors refer to those specific to a locality or site [14, 15] related to habitat, such as area size [16], heterogeneity [17], and connectivity [18], or the local environment [19, 20]. Intrinsic factors, or community factors [14] such as interactions between species (e.g., competition or facilitation), on the other hand, are generated by the community itself, or by the populations within the community [21]. Extrinsic factors influence species and genetic diversity directly, whereas intrinsic factors more often lead to a situation when two diversity levels causally affect one another [15]. For example, if the species diversity influences selection in the community [22], this can also affect the genetic diversity of species [15], or if genetic diversity determines the productivity of the community, there can be consequent effects on how much species diversity the community can support [1, 23]. Isolated communities and populations with reduced gene flow and low genetic diversity can exacerbate reductions in species diversity, since each species in the community is likely to go extinct by chance [15].

Both species and genetic diversity can be shaped by extrinsic factors, but all species in the community might not be equally susceptible to the same drivers. Species differ in their ecological function, life history, and tolerance to environmental dynamics. As a result, and because intrinsic factors are likely to be specific to particular species, it is reasonable to expect that not all taxa in a community show SGDCs in the same strength or even direction [24, 25]. Patterns that are observed for some species are not necessarily transferable to the remaining species, which is important to consider in conservation planning [26]. Since common species have a higher potential to resemble the surrounding community, positive SGDCs are expected to be more likely when the focal species is common, while rare or specialised species are more likely to show SGDC patterns that differ from other species in a community [4, 10, 24]. A multi-species approach, where genetic diversity is assessed in several focal species from the same community [27, 28], thus provides a more comprehensive insight into the importance of environmental dynamics to different species [14]. However, only few studies consider multiple species in a community when assessing SGDCs [10, 25, 28, 29], most likely due to the added complexity, costs and logistical challenges of multi-species studies.

Communities in the Baltic Sea are tractable for studying the relationship between species diversity and genetic diversity because they have low species and genetic diversity, and they are affected by significant extrinsic factors that could affect diversity levels. The Baltic Sea is characterised by its relatively young geological age, as well as its strong salinity gradient, from 35‰ at the entrance to the North Sea to 2‰ in the Gulf of Bothnia [30]. Unlike most marine environments that are typically characterised by high connectivity [6], dispersal in the Baltic Sea may be constrained for several reasons. Dispersal barriers originate not only from the oceanographic structure [31], but also from dispersal limitation due to local adaptation to low salinity [32], and differences in the dispersal potential of the species [33]. This combination of dispersal barriers as well as the ongoing processes of colonisation and invasion has shaped a distinctive species composition and community structure [34, 35]. The consequence of the above factors has been a decline in the species richness of benthic macrofauna with declining salinity, with the Gulf of Bothnia exhibiting about one tenth of the species richness compared to the North Sea [36, 37]. The changes in species diversity along the salinity gradient, is accompanied by a change in the species composition [38] with the most saline parts of the Baltic Sea dominated by marine taxa while freshwater taxa dominate the areas of low salinity [36]. Furthermore, marine fauna have invaded the Baltic Sea in several waves, and from different origins [39]. This has resulted in sibling species [40, 41], unique lineages of the same species [42], as well as cryptic species [43] occupying different areas of the Baltic Sea, all indicating low levels of connectivity of the populations. Dispersal barriers and, in particular, the salinity gradient not only affect species diversity, but also affect genetic diversity [44]. Many Baltic populations demonstrate a lower genetic diversity compared to Atlantic populations of the same species, which may indicate a bottleneck in gene flow between populations, sustained by the environmental gradients and the geographical isolation [44].

**Fig. 1:**
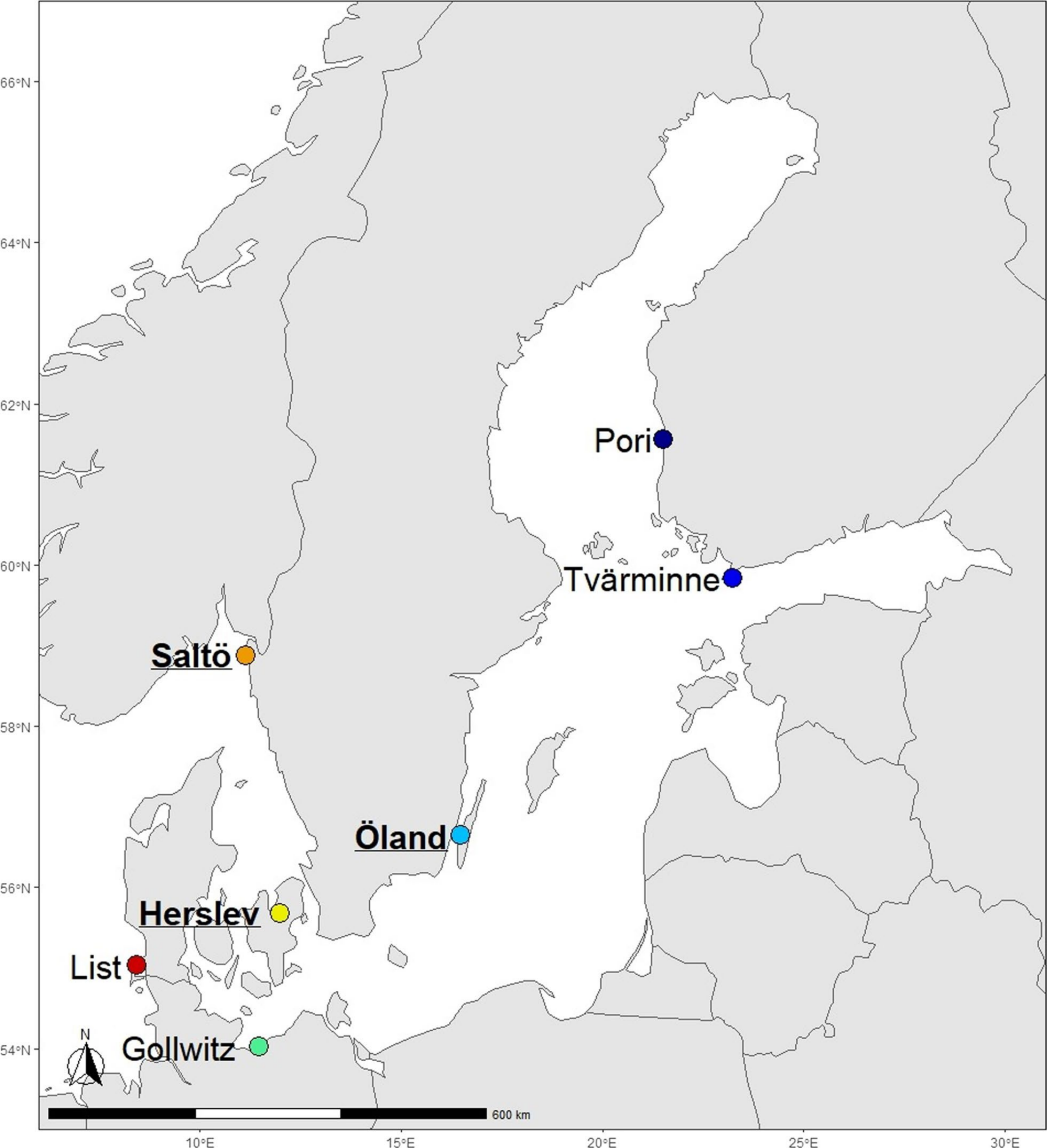
Map of the Baltic Sea, including sampling sites: List, Saltö, Herslev, Gollwitz, Öland, Tvärminne, Pori. Temporal sample sites Saltö, Herslev, Öland, indicated with bold and underlined

Using ultra-conserved elements (UCEs) and high throughput sequencing methods together with traditional species diversity quantification, we evaluate the species genetic diversity relationships between six macrofauna invertebrate species (*Hediste diversicolor*, *Pygospio elegans*, *Macoma balthica*, *Mya arenaria*, *Mytilus edulis*, and *Corophium volutator*) and the surrounding macrobenthic invertebrate community along the natural salinity gradient in the Baltic Sea and adjacent North Sea (Fig. 1). The six focal species exhibit wide zoogeographical distribution and relatively high abundance along the Baltic Sea salinity gradient [45] and therefore, should be good representatives of the macrobenthic invertebrate communities. However, the focal species also represent three taxonomic phyla (Annelida, Mollusca, and Arthropoda), with different classes exhibiting different ecological functions (see Table 1) and with different invasion histories [43, 46, 47]. Through assessing genetic diversity within several species with different attributes, the aim is to obtain a broad understanding of the potential community patterns in this ecosystem. Evaluation of the SGDCs together with several environmental variables both spatially and temporally, allows assessment of how specific environmental factors drive the SGDCs. It is hypothesised that salinity is a strong extrinsic factor in this estuarine habitat that has a strong effect on both species and genetic diversity, leading to positive SGDCs for all the focal species. Because the influence of salinity could be stronger for some species than others, however, SGDCs might not show significant relationships for all focal species.

**Table 1:** Taxonomic classification, reproduction strategy and ecological function of focal species

Species	Classification	Lifespan (years)	Larval type	Asexual reproduction	Life style	Feeding guild	Habitat type
*H. diversicolor*	Annelida Polychaeta Phyllodocida Nereididae	1–3	Benthic	No	Benthic Infauna Burrow dwelling	Omnivore Deposit feeder Predator	Mixed soft sediment
*P. elegans*	Annelida Polychaeta Spionida Spionidae	1–2	Benthic/ planktonic	Yes	Benthic Infauna Tube dwelling	Suspension feeder Filter feeder Deposit feeder	Sandy-muddy sediment Sheltered
*M. balthica*	Mollusca Bivalvia Cardiida Tellinidae	5–10	Planktonic	No	Benthic Infauna Burrow dwelling	Suspension feeder Deposit feeder	Soft sediments
*M. arenaria*	Mollusca Bivalvia Myida Myidae	10–12	Planktonic	No	Benthic Infauna Burrow dwelling	Suspension feeder	Soft sediments
*M. edulis*	Mollusca Bivalvia Mytilida Mytilidae	18–24	Planktonic	No	Benthic Epifauna Sessile	Suspension feeder	Hard substrate
*C. volutator*	Arthopoda Malacostraca Amphipoda Corophiidae	1	Benthic	No	Benthic Infauna Semi-permanent burrow dwelling Swim/crawl	Suspension feeder Deposit feeder	Soft to muddy sediment

## Results

### Species diversity

The spatial benthic macroinvertebrate dataset included a total of 8653 individuals, and the temporal dataset 18,996 individuals assigned to 73 taxa. In the spatial dataset average Shannon diversity ranged between 2.636 and 1.592, highest at List and lowest at Saltö, and in the temporal study it ranged between 4.082 and 1.212, highest at Saltö August 2019 and lowest at Herslev April 2019. In the spatial dataset *Tubificoides benedii* was the most abundant taxon in List, whereas Saltö, Herslev, and Gollwitz were dominated by *Hydrobia* spp., Öland had *Gammarus duebeni* in highest abundance*,* and at both Tvärminne and Pori, *M. balthica* was the most abundant species. In the temporal samples, *Hydrobia* spp. was always the most abundant taxon at Saltö and Herslev, apart from Saltö in April 2019 when *T. benedii* was most abundant. Öland in August 2018 was mostly dominated by *G. duebeni*, whereas *C. volutator* was most abundant in the November 2018 and April 2019 samples, and in August 2019 *Chironomida* spp. were most abundant. See Additional file 1: Table S1A and S1B for details.

### Genetic diversity

Not all targeted focal species were present in sufficient numbers (min. = 12) at all sampling sites and times to be included in the genetic analyses, even though they are represented in the community diversity data. Moreover, the focal species were found in different densities among the sampled sites and times, see Additional file 1: Tables S1A and S1B and Additional file 1: Table S2 for details. Average nucleotide diversity (π, the number of nucleotide differences per site in the UCE loci per sample pool) ranged between 0.0126 and 0.0019 (see Table 2) and the number of UCE loci from which nucleotide diversity was estimated ranged between 63 and 336 (see Table 3). The number of shared UCE loci, which are the loci within each species that are shared among all samples, was low, indicating high divergence among the samples. The low number of shared loci could, however, also have been influenced by low sequencing depth of the pools.

**Table 2:** Average nucleotide diversity (π) for each population

Sample	*H. diversicolor*	*P. elegans*	*M. balthica*	*M. arenaria*	*M. edulis*	*C. volutator*
List	0.0024	NA	0.0049	0.0035	0.0111	0.0034
Saltö Aug18	0.0038	0.0053	0.0057	0.0047	0.0050	–
Saltö Nov18	–	0.0045	0.0051	0.0049	0.0126	–
Saltö Apr19	0.0027	0.0046	0.0048	0.0037	0.0045	–
Saltö Aug18	0.0019	0.0068	–	0.0039	0.0056	–
Herslev Aug18	0.0024	0.0035	0.0046	0.0047	0.0035	0.0038
Herslev Nov18	0.0027	0.0049	0.0046	0.0050	0.0045	0.0036
Herslev Apr19	0.0024	0.0043	0.0062	0.0037	0.0098	0.0042
Herslev Aug19	0.0028	0.0061	0.0040	0.0040	0.0055	0.0025
Gollwitz	0.0023	0.0044	0.0048	0.0037	0.0055	0.0035
Öland Aug18	0.0028	–	0.0045	0.0038	0.0047	0.0031
Öland Nov18	0.0056	–	0.0025	0.0042	0.0041	0.0024
Öland Apr19	0.0032	–	0.0066	0.0054	0.0066	0.0026
Öland Aug19	0.0024	–	0.0043	0.0043	0.0031	0.0024
Tvärminne	0.0023	–	0.0060	–	0.0072	0.0029
Pori	0.0035	–	0.0053	–	–	–

“–” indicates samples not included in the study due to absence or too few individuals available to include in genetic diversity analysis. “NA” indicates samples with missing value due to technical problems in sequencing

**Table 3:** Range of total number of amplified UCE loci for each species in all samples, and total number of shared UCE loci between all samples for each species

Species	Number of UCE loci	Number of shared UCE loci
*H. diversicolor*	116–308	6
*P. elegans*	85–199	14
*M. balthica*	56–249	1
*M. arenaria*	110–272	29
*M. edulis*	63–186	4
*C. volutator*	93–336	19

### SGDCs and environmental drivers

**Fig. 2:**
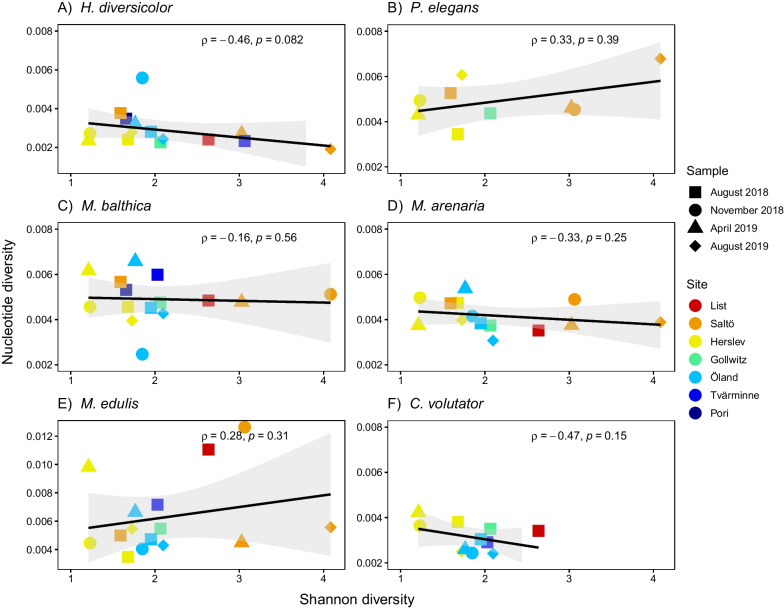
Relationship between average species diversity (Shannon) and nucleotide diversity (π) for the individual species, including both spatial and temporal samples. **A** *Hediste diversicolor*, **B** *Pygospio elegans*, **C** *Macoma balthica*, **D** *Mya arenaria*, **E** *Mytilus edulis* (note that y-axis for this plot is not the same as the others), **F** *Corophium volutator*. Colours indicating site and different symbols indicate sample time. Correlation coefficients based on Spearman’s correlation; line tendencies were obtained by regressions

Examining SGDCs between species diversity and genetic diversity for each focal species showed differing patterns. None of the SGDCs were statistically significant. Negative trends in SGDCs were observed for *H. diversicolor, M. balthica, M. arenaria,* and *C. volutator* (Fig. 2A, C, D, F) and positive trends were observed for *P. elegans* and *M. edulis* (Fig. 2B, E).

Measures of environmental variables for both spatial and temporal samplings are reported in detail in Table 4. When analysing the role of specific environmental parameters using a generalised linear mixed model (GLMM), random effects of site explained a large proportion of the variation for nucleotide diversity (see Additional file 1: Table S3, intercept estimates). This indicates that the patterns for nucleotide diversity measures are shaped by parameters or processes not included in the present study. The only significant relationship between an environmental variable and Shannon diversity was with C/N ratio for *M. balthica* (p = 0.040); otherwise there were no significant relationships between species diversity and the measured environmental variables for the SGDC datasets for any of the species studied. Environmental variables were more often related to genetic diversity. There was a significant relation between genetic diversity and the environmental factors for *M. balthica*, which was positively related to temperature (p = 0.048), and for *M. edulis,* which was positively related to salinity (p = 0.003), C/N ratio (p = 0.050) and sorting (p = 0.025). The discrepancy between datasets (both diversity measures and focal species datasets), might be due to the different sample points between the datasets and few sample points overall. For details of GLMM analysis see Additional file 1: Table S3.

**Table 4:** Environmental variables measured at all sample sites and times

Site	Sample time	Salinity	Temp, °C	C/N ratio	Organic matter, mg	Water-content, %	Porosity, %	Mean grain size, ϕ	Sorting (σ_I_)
List	August 2018	33	22	9.48	0.940	17.59	0.34	1.41	1.27
Saltö	August 2018	26	18	10.46	1.16	24.03	0.43	2.24	1.76
	November 2018	25	3	8.86	1.24	27.22	0.50	2.82	0.82
	April 2019	23	11	8.57	1.07	21.20	0.40	2.35	1.53
	August 2019	22	18	7.77	1.34	25.17	0.47	2.16	1.90
Herslev	August 2018	15	23	6.31	1.06	21.56	0.40	1.37	0.70
	November 2018	16	5	4.40	1.16	23.35	0.48	2.15	0.85
	April 2019	15	15	1.94	1.02	22.46	0.40	2.35	0.85
	August 2019	15	17	8.30	1.23	20.37	0.37	2.06	1.05
Gollwitz	August 2018	13	23	9.83	1.42	27.63	0.49	2.78	0.89
Öland	August 2018	7	21	9.89	0.89	22.69	0.43	2.40	0.63
	November 2018	8	1	3.83	0.61	24.15	0.44	2.70	0.41
	April 2019	9	14	7.06	0.55	23.42	0.44	2.50	0.46
	August 2019	8	18	6.90	0.70	20.75	0.37	2.48	0.49
Tvärminne	August 2018	7	13	6.82	1.40	32.41	0.56	2.60	0.67
Pori	August 2018	6	18	8.64	0.24	22.42	0.40	2.49	0.63

## Discussion

This study investigated species-genetic diversity correlations (SGDCs) in benthic invertebrate communities of the Baltic Sea. Analyses were carried out for six focal species sampled over a large spatial scale in the Baltic Sea and the adjacent North Sea, as well as on a seasonal temporal scale in a subset of the communities. In addition, the association of selected environmental factors with both species and genetic diversity was also investigated. Assuming ecological similarity, focal species that are common are expected to show positive SGDCs, while rare species are more likely to differ from the overall community [4]. Although the studied focal species are common throughout the Baltic Sea on a broad scale, we observed variation in their abundance, and even absences of some species at specific study sites. The variation occurred both in space, due to their natural distributions, and in time, which may be due to environmental events, such as heat waves, or other natural seasonal fluctuations. There were both negative and positive SGDC trends for the individual focal species, but none of them were statistically significant. This shows that even though species are common and widely distributed, they may differ ecologically from the overall community, and will not necessarily have a positive SGDC. Lino et al. [48] made a similar observation in their study of neotropical bats. Species that showed positive trends in SGDCs in our study tended to have lower densities at sampling sites with low species diversity, while species with negative trends in SGDCs typically had higher densities at all sites and times. Even species from the same taxonomic class (e.g., Annelids) displayed SGDCs with different trends, suggesting that species-specific differences are important and cannot be ignored when analysing the relationship between species and genetic diversity. Moreover, different environmental factors were associated with the genetic diversity of the individual species, while C/N ratio was the only environmental variable associated with species diversity and for only one species dataset, even though it is known that alpha-diversity in the Baltic sea is affected by salinity [37].

Many studies of SGDCs consider a single focal species [6, 49–52], which is expected to represent the entire community. Including several focal species in this study was, in contrast, advantageous, because it revealed different trends among the species, highlighting that even common and dominant members of the benthic community are not necessarily representative of other species. On the other hand, inclusion of multiple focal species had the disadvantage that it restricted the scope of the study to only a few sites, which might not, in hindsight, have been enough for detecting the SGDCs. Considering that salinity was surprisingly not found to be associated with species diversity in this study, including more study sites across the large spatial range was likely needed to capture the full range of variation in community species diversity in the Baltic Sea. Previous studies considering multiple species revealed positive SGDCs over large geographical scales than we studied [53–56] that were driven by larger scale extrinsic forces such as isolation, habitat area, climate and historical processes. Another difference in those studies compared to our study is that genetic diversity was measured in different species from the same taxonomic class and order. Species belonging to the same class might be more similar in respect to how environmental drivers affect their diversity, and thus, more likely to result in SGDCs with the same directionality.

Ecological similarity among species is highlighted in the theory explaining SGDCs [14], and it was expected that if focal species are representative of the rest of the community in regards to their ecology, a positive SGDC would result. Of course, benthic invertebrate species have many differences in ecological properties, such as differences in feeding guilds and lifestyle, also within a single taxonomic class (see Table 1) which makes the choice of representative focal species challenging. For example, the sediment-burrowing focal species (*H. diversicolor*, *P. elegans*, *M. arenaria*, *M. balthica*, and *C. volutator*, see Table 1) might be expected to respond to the studied sediment variables (porosity, mean grain size and sorting) in a similar way, since sediment properties and bioavailability of nutrients are known to be important environmental factors that shape benthic communities [57]. However, this was not the case, and sorting only had a significant effect on genetic diversity of *M. edulis,* an epifaunal species not living directly in contact with the sediment. The measured variables C/N ratio and organic matter may represent bioavailability of nutrients in both the sediment and water column to some degree. Therefore, both suspension and deposit feeders in the community could respond in the same way to these variables. However, only the genetic diversity of the suspension feeding *M. edulis* was affected by C/N ratio, a relation which was negative. In addition, there were no shared trends in the directionality of the SGDCs between species of same feeding guild. More work is needed to understand to which degree ecological roles, such as feeding guilds, dictate patterns of SGDCs in these communities.

The life histories and life spans of different species in the community could play a role in determining the diversity patterns. Due to recruitment events, population dynamics of marine invertebrates are likely to be closely tied to seasonal change in temperature [58], and temperature can be used as a proxy for variation in production and reproductive patterns for communities in seasonally variable environments, where reproduction takes place in spring or summer. Only the genetic diversity of *M. balthica* was positively related to temperature, hence seasonality. Like many other marine organisms, this bivalve is known to time their reproduction to temperature and seasonal events [59, 60]. A positive SGDC linked to temperature was found for *P. elegans* previously, which was explained by its reproductive patterns and connectivity among populations showing genetic differences among cohorts of worms settling in the populations at different times [6], but in the present study, there was no significant effect of temperature on genetic diversity of *P. elegans*, nor any indication that the other measured environmental variables affect the SGDC of *P. elegans*. This suggests that the reported environmental variables have not captured the full extent of possible extrinsic drivers of diversity of *P. elegans* or that the current study does not have sufficient statistical power to reveal a SGDC due to the relatively small sample size.

Although benthic invertebrate communities of the Baltic Sea might be tractable for investigating potential SGDCs due to their low diversity and the salinity gradient, their varied and still changing population histories could have influenced our result and led to the lack of correlations between species diversity and genetic diversity. Many species show declines in genetic diversity along the salinity gradient in the Baltic Sea [44], but the patterns can be complicated, resulting from the different invasion histories of the various taxa [61]. For example, *H. diversicolor* exists as a species complex of two cryptic species that are reproductively isolated and partly sympatric [43]. The subspecies more prevalent in the northern Baltic Sea generally has high genetic diversity ([61] and this study), and has shown evidence of adaptation to the low salinity, while the subspecies in the southern part of the Baltic Sea is less diverse and possibly the result of a more recent colonisation [43, 62]. The history of different invasion events and mixing of divergent lineages of *H. diversicolor* in the northern Baltic Sea could explain why a negative trend in SGDC for this species was found. Likewise, *M. balthica* has invaded the Baltic Sea multiple times, from both the Atlantic and the Pacific, resulting in a hybrid swarm and genetically unique lineages [47, 63]. Hybridisation between the different subspecies could lead to higher genetic diversity of *M. balthica* in the inner Baltic Sea compared to the North Sea [47]. The *M. edulis/trossulus* complex also includes different subspecies inhabiting different basins of the Baltic Sea [40, 41]. Although the salinity gradient in the Baltic is a potential environmental driver of diversity patterns, the historical patterns of diversity determined by different invasion histories may have a stronger influence on some species. Although all focal species are of marine origin, they are also all well-established species in estuarine and brackish water ecosystems, including the Baltic Sea [45, 64]. Therefore, it is likely that they are adapted to the low salinity regime to such a degree that low salinity is not a significant stressor on their populations [65–67]. Their wide salinity tolerance raises the question of whether salinity is a significant determinant of diversity patterns for these species, in contrast to the original hypothesis formulated here. Salinity is, however, a likely driver for community diversity given that the number of marine species that can be present falls with lower salinity.

It is also important to consider the context, and the life history of the focal species when assessing SGDCs. Since genetic diversity in species with short life spans can have seasonal fluctuations [68], spatial SGDCs based on such species could lead to non-significant results, no SGDC or even skewed conclusions, raising the question if the theory is appropriate for these species. SGDCs assessed using species with annual lifecycles on a temporal scale will, however, provide information about responses to seasonal environmental changes and population dynamics. On the other hand, measuring genetic diversity in individuals of different age classes in species with long life spans will provide a better understanding of large-scale drivers of SGDCs such as adaptation to environmental clines, large scale population connectivity, and invasion histories.

The present study aimed to evaluate species-genetic diversity correlations from a range of focal species that are common and potentially show ecological similarity with the diverse benthic invertebrate community. To do so, genetic diversity was estimated based on nucleotide diversity of sequences from ultra-conserved elements. Ultra-conserved elements have previously been used for phylogeographic- and population genetic studies [69–72]. Potentially, ultra-conserved elements can also be found among phyla [73], so that diversity can be estimated from comparable loci in evolutionary distant taxa. This is, however, more challenging, as more mutations may have accumulated with longer divergence times. We were unsuccessful in finding shared loci among all the studied species and sites for an analysis of SGDC that could incorporate all focal species together. Even within species, the low number of shared loci among the different sites raises questions of the effectiveness of the UCEs for estimates of genetic diversity in studies of SGDCs. This could have also been the result of the differing population histories and isolation of populations in the Baltic Sea.

## Conclusions

Based on this study, it is unrealistic to expect that common species, and the community as a whole, to respond in the same way to environmental factors, and thus show a positive SGDCs. Therefore, extrapolation of drivers of community processes based on measurements from only a few common species is questionable. This emphasises the weakness of using only one or few species as representatives for a taxonomic group or as an indicator species for a community in, for example, conservation assessments [26]. The genetic history of the species and populations in the Baltic Sea, which stems from their invasion histories and unique adaptation to the environment in this brackish water system, may explain the differing patterns of diversity among the present samples. The results highlight the importance of the choice of focal species when assessing SGDCs and emphasise the importance of including a wide range of species, also from the same taxonomic order or class, when assessing and implementing conservation management plans based on genetic diversity assessments [12]. Although earlier studies of SGDCs have focused on positive SGDCs [14], negative correlations and even absence of relationships revealed in this study, are important for understanding diversity patterns of the individual species, moreover, the interactions between the species in the community and the drivers affect the diversity.

## Methods

### Sampling macrofauna and environmental data

Sampling on a spatial scale was carried out during August 2018, at seven study sites (List, Saltö, Herslev, Gollwitz, Öland, Tvärminne, Pori) in the North Sea and Baltic Sea (Fig. 1), and continued on a temporal scale at four times during a year (August 2018, November 2018, April 2019, August 2019) at three of the sampling sites (Saltö, Herslev, Öland) in the Baltic Sea (Fig. 1, stations indicated with bold and underline). All sites were sampled from the coast at 0–0.80 m water depth, except for Tvärminne, where sampling was also performed by SCUBA at 3.8–5 m depth.

Five replicate sediment cores were collected at each site and time using hand-held corers (15 cm diameter, 30 cm depth). Samples were sieved through a 1 mm mesh, and remaining material was fixed in 99% ethanol on site. In the laboratory, specimens were sorted and identified to the lowest reliable taxonomic level according to Kirkegaard [74], Kirkegaard [75], Barnes [76], and Hayward and Ryland [77], and taxonomic validity confirmed in World Register of Marine Species [78]. Sorted specimens were stored in 99% ethanol. After quantitative core sampling, a further 12–20 specimens of the six benthic macrofauna focal species (the polychaete worms *H. diversicolor* and *P. elegans,* the bivalves *M. Balthica, M. arenaria, M. edulis,* and the crustacean *C. volutator*) were collected (if present) for assessing genetic diversity within the species (see Additional file 1: Table S2). Due to its absence at the original sampling site, *C. volutator* was always sampled approximately 3 km from the Herslev sampling site*.* All specimens were stored in 99% ethanol for later analysis. Species identity was confirmed in the laboratory post collection, using a dissecting microscope (20× magnification) and/or COI molecular barcoding tools.

Sediment characteristics (including water content, porosity, organic content, carbon and nitrogen content, grain size and sorting) were determined from three replicate cores per sampling site (5 cm diameter, min. 15 cm depth). In the laboratory, the top 2 cm of the three cores were pooled and mixed before analysis. Wet weight and dry weight (24 h at 105 °C) of 5 cm^3^ of the mixed top sediment was used to determine water content and porosity. Organic content was determined from loss on ignition (2 h at 550 °C) of 5 cm^3^ dried sediment. For carbon and nitrogen content, two replicate samples were dried and pre-combusted at 550 °C for 2 h to remove organic C before analysing C content to correct for CaCO_3_ from e.g., shells in the sediment. Pulverised sediment samples (30–50 mg) were analysed using element analyser Flash 2000 NCS-Analyzer and FlashEA® 1112 CHNO Analyzer, Thermo Scientific. Organic C was calculated as the difference between total C from samples minus carbonate C from pre-combusted samples. Particle size was determined by the proportion of dry weight of each size fraction of the Wentworth size scale (4 mm, 2 mm, 1 mm, 0.5 mm, 0.25 mm, 0.125 mm, 0.063 mm), by wet sieving 50–150 g wet sediment by hand. Median particle size and sediment sorting (σ_I_, with lower sorting values reflecting more well sorted sediment, and higher numbers more poorly sorted sediment) were calculated from logarithmic particle size (ϕ) using the Folk and Ward method in GRADISTAT v. 9.1 [79]. Temperature was measured using handheld field thermometer (Frederiksen Scientific), salinity was measured using ATAGO handheld refractometer (resolution of 0.5 practical salinity units, PSU) in the field.

### DNA extraction, UCE library preparation and sequencing

DNA was extracted using the DNeasy Blood & Tissue Kit (Qiagen). For small specimens (*P. elegans, M. arenaria, M. balthica, C. volutator*) DNA was extracted from whole complete specimens; for large individuals, DNA was extracted from heads (*H. diversicolor*) or foot tissue (*M. edulis, M. arenaria, M. balthica*). DNA concentration was quantified using a Qubit 4.0 fluorometer with 1X dsDNA HS Assay Kit (Thermo Fisher, Cambridge, UK). For library preparations and sequencing, 12–20 individuals were pooled with equal concentration in populations (separate pools for each species and sampling site/time). Pools were purified using the QIAquick Nucleotide Removal Kit (Qiagen). Previously, UCE loci were identified and a probe set for all the targeted taxonomic groups was developed using reference genomes (Additional file 1: Table S4) as described in Petersen et al. [80], the probe set is available at JYX Digital Repository [81]. The UCE probe set was used to capture the desired loci from the present DNA pools (UCE library), which were prepared for amplification and sequencing using standard protocols at Arbor Biosciences (Ann Arbor, MI, USA, arborbiosci.com), briefly described below.

At Arbor Biosciences, DNA pool samples were sonicated, and size selected to an average insert length of approximately 500 nt. For UCE capture, up to 200 ng DNA of each pool was used for library preparation, and unique dual-index combinations were added to each sample via 9 cycles of PCR amplification. Indexed libraries were quantified with both a spectrofluorometric assay and quantitative PCR. Up to 1 µg (or 80% of the library volume if 1 µg was not available) was dried down to 7 µL by vacuum centrifugation. UCE capture was performed following the myBaits v5 protocol with overnight hybridization at 65 °C and washes at 65 °C. For each, half of the volume of beads in the elution buffer were amplified for eight cycles and the second half of the beads were amplified for 14 cycles. The two halves were combined and quantified with both a spectrofluorometric assay and a quantitative PCR assay. UCE captures of the different samples were combined in approximately equimolar ratios, but some captures were underrepresented due to lack of DNA availability. A screen using a MiSeq Nano PE150 sequencing run was performed to check equilibration. Samples were then sequenced on the Illumina NovaSeq 6000 platform on a partial S4 PE150 lane with v1.5 chemistry. Due to a low number of reads in the initial sequencing run, a second sequencing run was performed, and demultiplexed reads for each sample from both runs were combined.

### Bioinformatic analyses

Raw demultiplexed sequencing reads were length trimmed to a minimum length 40 bp, adapter trimmed and quality trimmed to a phred score of 33, using illumiprocessor v. 2.10 with trimmomatic v. 0.39 [82–84] (https://github.com/faircloth-lab/illumiprocessor)*.* Following trimming, the reference UCE loci for each sample were prepared. Firstly, sequence reads from each sample pool were assembled into contigs using ABySS in phyluce v. 1.7.0 [73, 85, 86] (https://github.com/faircloth-lab/phyluce) with a kmer of 35. Contigs were then aligned to the UCE probe set [81] with a minimum coverage and identity 80, while removing duplicates using phyluce’s integration of LASTZ v. 1.04.00 [87]. Based on the contig assembly, an incomplete matrix for the UCE loci for all samples was produced (since no UCE loci were shared between all taxa), and a separate FASTA file of UCE loci for each sample was generated from this matrix and extracted using phyluce. A complete matrix for each taxon was made to calculate the number of shared loci per taxon. The trimmed Illumina reads of each sample were then mapped to their respective UCE locus FASTA file separately, and subsequently paired using BWA v. 0.7.17 [88] (https://github.com/lh3/bwa). The mapped paired read files were filtered to keep only proper pairs, removing unmapped reads and reads with unmapped mates, and formatted to a BAM-file using samtools v. 1.10 [89] (https://github.com/samtools/samtools). After removal of duplicates using sambamba v. 0.8.0 [90] (https://github.com/biod/sambamba), individual BAM-files were sorted and converted to pileup files using samtools, and indels removed using PoPoolation 1.2.2 [91] (https://sourceforge.net/projects/popoolation). Mapping of single-nucleotide polymorphisms (SNPs) in each sample pool to their respective UCE loci based on the incomplete matrix, and calculation of nucleotide diversity (π), number of nucleotide differences per site, was done over a sliding window using PoPoolation, with parameters: window and step size 1000, min. covered fraction 0.1, min. count 1, min. coverage 2, max. coverage 20, and a pool size of 24–40, according to the original pool size multiplied by two to correct for diploidy. See Additional file 1: Fig. S1 for illustration of the bioinformatic process.

### Statistical analysis

Abundance of each identified taxon at each sampling site/time was recorded in the software PRIMER-e v.7.0.20 [92] and species diversity was calculated as Shannon diversity (calculated by log e) for each replicate, and averaged over replicates. Genetic diversity estimates were based on calculations of nucleotide diversity (π) as described above, for each locus and window of 1000 bp and averaged over all loci per sample pool. The SGDC was calculated between Shannon diversity and nucleotide diversity (π), using Spearman’s rank correlation coefficient in R version 3.6.3 (R Core Team 2020). We calculated SGDCs for the focal species separately using both spatial and temporal data, to secure a minimum of sampling points. We used a Durbin-Watson test to ensure that the temporal samples were not autocorrelated, implemented in the R package car v. 3.0–10 [94]. All plots were made using ggplot2, v. 3.3.0 [95].

The present hypothesis was that diversity within all the focal species and benthic invertebrate community would be driven by predominant environmental factors. Environmental impact on Shannon diversity measures was assessed using generalised linear mixed models (GLMM). Eight environmental variables were included in the analysis: salinity, sediment mean grain size, sorting, sediment water content, porosity, C/N ratio, organic content, and sediment temperature. Since the sediment variables are likely to co-vary, testing for collinearity between them using scatterplots and Pearson correlation coefficient was conducted. There was a strong correlation between sediment porosity and water content (r = 0.95); hence, to reduce the number of explanatory variables it was decided to remove water content from the analysis, keeping porosity for further analysis.

To only assess the environmental impact of the communities with the focal species present, GLMM for Shannon diversity was performed on subset datasets including only sample sites where the focal species was present for genetic diversity. The distribution of species diversity dataset was tested and visualised prior to analysis using R package vcd v. 1.4–8 [96], the Poisson distribution was the best fitting model. As a random effect, a sample was included, representing a combination of sampling site and time, to account for effects of repeated measures of the present temporal samples. The GLMM was performed with glmmPQL in the R package MASS v. 7.3–54 [97] as follows:

$$\begin{aligned} {\text{Log}}\left( {\text{Species Diversity}} \right) \, & = \, \alpha \, + \, \beta {1 } \times {\text{ mean grain size }} + \, \beta {2 } \times {\text{ sorting }} + \, \beta {3 } \times {\text{ salinity }} + \, \beta {4 } \times {\text{ porosity }} + \, \beta {5 } \times {\text{ C}}/{\text{N ratio }} \\ & \quad + \, \beta {6 } \times {\text{ organic content }} + \, \beta {7 } \times {\text{ temperature }} + {\text{ Poisson}}\left( {\lambda {\text{Sample}}} \right) \, + {\text{ Poisson}}\left( {\lambda {\text{Residual}}} \right) \\ \end{aligned}$$

Nucleotide diversity, π, is neither count data nor is normally distributed, therefore a quantile comparison plot using R package car was consulted, and it found the best fit to a log-normal distribution. A log-linear model with a normally distributed error term was used to test the effect of environmental variables. The same fixed effects were used as described above, however, including only site as a random effect due to lack of replication within sample:

$$\begin{aligned} {\text{Log}}\left( {\text{Nucleotide diversity}} \right) \, & = \, \alpha \, + \, \beta {1 } \times {\text{ mean grain size }} + \, \beta {2 } \times {\text{ sorting }} + \, \beta {3 } \times {\text{ salinity }} \\ & \quad + \, \beta {4 } \times {\text{ porosity }} + \, \beta {5 } \times {\text{ C}}/{\text{N ratio }} + \, \beta {6 } \times {\text{ organic content }} \\ & \quad + \, \beta {7 } \times {\text{ temperature }} + {\text{ Normal}}\left( {0,\sigma {\text{2 Site}}} \right) \, + {\text{ Normal}}\left( {0,\sigma {\text{2 Residual}}} \right) \\ \end{aligned}$$

## Supplementary Information

**Additional file 1.** Raw species diversity data, full results of GLMM analyses, additional data for probe design, and overview of bioinformatic procedure.

## Data Availability

The UCE probe set is available from JYX data repository [81], and other datasets used and/or analysed during the current study are available from the corresponding author on request.
