# Supplementary material for: Species and genetic diversity relationships in benthic macroinvertebrate communities along a salinity gradient
Source: BMC Ecol Evol. 2022 Nov 2;22:125. doi: 10.1186/s12862-022-02087-6 (PMC9632067; doi:10.1186/s12862-022-02087-6)
Supplement: Supplementary file 1 — Additional file 1. Raw species diversity data, full results of GLMM analyses, additional data for probe design, and overview of bioinformatic procedure. [file 12862_2022_2087_MOESM1_ESM.docx]

Supplementary table 1A: Results of quantitative species diversity analysis, spatial study. Number of individuals of each species pr. replicate core (706,5 cm^2^). Abbreviations for sites L (List), S (Saltö), H (Herslev), G (Gollwitz), Ö (Öland), T (Tvärminne), P (Pori), each site is represented by 5 replicates.

| **Species** | **L1** | **L2** | **L3** | **L4** | **L5** | **S1** | **S2** | **S3** | **S4** | **S5** | **H1** | **H2** | **H3** | **H4** | **H5** | **G1** | **G2** | **G3** | **G4** | **G5** | **Ö1** | **Ö2** | **Ö3** | **Ö4** | **Ö5** | **T1** | **T2** | **T3** | **T4** | **T5** | **P1** | **P2** | **P3** | **P4** | **P5** |
| --- | --- | --- | --- | --- | --- | --- | --- | --- | --- | --- | --- | --- | --- | --- | --- | --- | --- | --- | --- | --- | --- | --- | --- | --- | --- | --- | --- | --- | --- | --- | --- | --- | --- | --- | --- |
| **Annelida** |  |  |  |  |  |  |  |  |  |  |  |  |  |  |  |  |  |  |  |  |  |  |  |  |  |  |  |  |  |  |  |  |  |  |  |
| Alitta succinea | 0 | 0 | 0 | 0 | 0 | 0 | 0 | 0 | 0 | 0 | 0 | 0 | 0 | 0 | 0 | 0 | 0 | 0 | 1 | 0 | 0 | 0 | 0 | 0 | 0 | 0 | 0 | 0 | 0 | 0 | 0 | 0 | 0 | 0 | 0 |
| Arenicola marina | 1 | 0 | 0 | 1 | 0 | 0 | 0 | 0 | 3 | 0 | 0 | 0 | 0 | 0 | 0 | 0 | 0 | 0 | 0 | 0 | 0 | 0 | 0 | 0 | 0 | 0 | 0 | 0 | 0 | 0 | 0 | 0 | 0 | 0 | 0 |
| Capitella capitata | 0 | 2 | 0 | 0 | 0 | 0 | 0 | 0 | 0 | 0 | 0 | 0 | 0 | 0 | 0 | 1 | 0 | 0 | 0 | 0 | 0 | 0 | 0 | 0 | 0 | 0 | 0 | 0 | 0 | 0 | 0 | 0 | 0 | 0 | 0 |
| Capitella giardi | 3 | 0 | 0 | 1 | 0 | 0 | 0 | 0 | 0 | 0 | 0 | 0 | 0 | 0 | 0 | 1 | 0 | 0 | 0 | 0 | 0 | 0 | 0 | 0 | 0 | 0 | 0 | 0 | 0 | 0 | 0 | 0 | 0 | 0 | 0 |
| Capitellidae sp. | 0 | 0 | 17 | 0 | 3 | 0 | 0 | 0 | 0 | 0 | 0 | 0 | 0 | 0 | 0 | 0 | 0 | 0 | 3 | 0 | 0 | 0 | 0 | 1 | 0 | 0 | 0 | 0 | 0 | 0 | 0 | 0 | 0 | 0 | 0 |
| Chaetozone setosa | 20 | 3 | 32 | 36 | 0 | 0 | 0 | 0 | 0 | 0 | 0 | 0 | 0 | 0 | 0 | 0 | 0 | 0 | 0 | 0 | 0 | 0 | 0 | 0 | 0 | 0 | 0 | 0 | 0 | 0 | 0 | 0 | 0 | 0 | 0 |
| Cirratulidae spp. | 0 | 0 | 0 | 1 | 4 | 0 | 0 | 0 | 0 | 0 | 0 | 0 | 0 | 0 | 0 | 0 | 0 | 0 | 0 | 0 | 0 | 0 | 0 | 0 | 0 | 0 | 0 | 0 | 0 | 0 | 0 | 0 | 0 | 0 | 0 |
| Cirratulus cirratulus | 0 | 0 | 0 | 3 | 3 | 0 | 0 | 0 | 0 | 0 | 0 | 0 | 0 | 0 | 0 | 0 | 0 | 0 | 0 | 0 | 0 | 0 | 0 | 0 | 0 | 0 | 0 | 0 | 0 | 0 | 0 | 0 | 0 | 0 | 0 |
| Dipolydora coeca | 0 | 0 | 0 | 2 | 0 | 0 | 0 | 0 | 0 | 0 | 0 | 0 | 0 | 0 | 0 | 0 | 0 | 0 | 0 | 0 | 0 | 0 | 0 | 0 | 0 | 0 | 0 | 0 | 0 | 0 | 0 | 0 | 0 | 0 | 0 |
| Eteone barbata | 0 | 0 | 2 | 0 | 0 | 0 | 0 | 0 | 0 | 0 | 0 | 0 | 0 | 0 | 0 | 0 | 0 | 0 | 0 | 0 | 0 | 0 | 0 | 0 | 0 | 0 | 0 | 0 | 0 | 0 | 0 | 0 | 0 | 0 | 0 |
| Eteone longa | 0 | 1 | 0 | 0 | 0 | 0 | 0 | 0 | 0 | 0 | 0 | 0 | 0 | 0 | 0 | 0 | 0 | 0 | 0 | 0 | 0 | 0 | 0 | 0 | 0 | 0 | 0 | 0 | 0 | 0 | 0 | 0 | 0 | 0 | 0 |
| Eteone sp. | 0 | 0 | 0 | 1 | 0 | 0 | 0 | 0 | 0 | 0 | 0 | 0 | 0 | 0 | 0 | 0 | 0 | 0 | 0 | 0 | 0 | 0 | 0 | 0 | 0 | 0 | 0 | 0 | 0 | 0 | 0 | 0 | 0 | 0 | 0 |
| Hediste diversicolor | 7 | 3 | 5 | 6 | 6 | 33 | 7 | 7 | 2 | 0 | 11 | 31 | 30 | 43 | 14 | 9 | 52 | 60 | 39 | 37 | 38 | 7 | 22 | 31 | 16 | 4 | 4 | 7 | 2 | 2 | 8 | 1 | 0 | 12 | 0 |
| Heteromastus filiformis | 6 | 4 | 8 | 3 | 0 | 0 | 0 | 0 | 0 | 4 | 0 | 0 | 0 | 0 | 0 | 1 | 0 | 4 | 0 | 0 | 0 | 0 | 0 | 0 | 0 | 0 | 0 | 0 | 0 | 0 | 0 | 0 | 0 | 0 | 0 |
| Marenzelleria spp. | 0 | 1 | 0 | 0 | 0 | 0 | 0 | 0 | 1 | 0 | 0 | 0 | 0 | 0 | 0 | 0 | 0 | 0 | 0 | 0 | 0 | 1 | 1 | 2 | 1 | 29 | 23 | 13 | 10 | 7 | 0 | 11 | 15 | 3 | 0 |
| Mediomastus fragilis | 0 | 0 | 0 | 0 | 0 | 0 | 0 | 0 | 0 | 1 | 0 | 0 | 0 | 0 | 0 | 1 | 0 | 0 | 0 | 0 | 0 | 0 | 0 | 0 | 0 | 0 | 0 | 0 | 0 | 0 | 0 | 0 | 0 | 0 | 0 |
| Nereis virens | 0 | 0 | 0 | 0 | 1 | 1 | 0 | 0 | 0 | 0 | 0 | 0 | 0 | 0 | 0 | 0 | 0 | 0 | 0 | 0 | 0 | 0 | 0 | 0 | 0 | 0 | 0 | 0 | 0 | 0 | 0 | 0 | 0 | 0 | 0 |
| Nerilla antennata | 7 | 0 | 0 | 0 | 0 | 0 | 0 | 0 | 0 | 0 | 0 | 0 | 0 | 0 | 0 | 0 | 0 | 0 | 0 | 0 | 1 | 0 | 0 | 0 | 0 | 0 | 0 | 0 | 0 | 0 | 0 | 0 | 0 | 0 | 0 |
| Phyllodoce maculata | 0 | 0 | 0 | 2 | 0 | 0 | 0 | 0 | 0 | 0 | 0 | 0 | 0 | 0 | 0 | 0 | 0 | 0 | 0 | 0 | 0 | 0 | 0 | 0 | 0 | 0 | 0 | 0 | 0 | 0 | 0 | 0 | 0 | 0 | 0 |
| Polydora ciliata | 0 | 0 | 0 | 2 | 0 | 0 | 0 | 0 | 0 | 0 | 0 | 0 | 0 | 0 | 0 | 0 | 0 | 0 | 0 | 0 | 0 | 0 | 0 | 0 | 0 | 0 | 0 | 0 | 0 | 0 | 0 | 0 | 0 | 0 | 0 |
| Polydora cornuta | 1 | 0 | 1 | 0 | 0 | 1 | 0 | 0 | 0 | 0 | 0 | 0 | 0 | 0 | 0 | 0 | 0 | 4 | 0 | 0 | 0 | 0 | 0 | 0 | 0 | 0 | 0 | 0 | 0 | 0 | 1 | 0 | 0 | 0 | 0 |
| Polydora spp. | 0 | 0 | 1 | 2 | 0 | 0 | 0 | 2 | 0 | 0 | 0 | 0 | 0 | 0 | 0 | 0 | 0 | 0 | 0 | 0 | 0 | 0 | 0 | 0 | 0 | 0 | 0 | 0 | 0 | 0 | 0 | 0 | 0 | 0 | 0 |
| Pygospio elegans | 2 | 7 | 17 | 7 | 3 | 4 | 0 | 0 | 1 | 0 | 0 | 0 | 4 | 5 | 0 | 0 | 0 | 0 | 0 | 0 | 0 | 0 | 0 | 0 | 0 | 0 | 0 | 0 | 0 | 14 | 0 | 0 | 0 | 0 | 0 |
| Streblospio shrubsolii | 0 | 0 | 0 | 2 | 0 | 0 | 0 | 0 | 0 | 0 | 0 | 0 | 0 | 0 | 0 | 0 | 0 | 0 | 0 | 0 | 0 | 0 | 0 | 0 | 0 | 0 | 0 | 0 | 0 | 0 | 0 | 0 | 0 | 0 | 0 |
| Enchytraeidae sp. | 3 | 0 | 5 | 0 | 0 | 0 | 0 | 0 | 0 | 0 | 0 | 14 | 0 | 0 | 0 | 0 | 0 | 22 | 0 | 0 | 0 | 1 | 0 | 0 | 0 | 0 | 0 | 0 | 0 | 0 | 0 | 0 | 0 | 0 | 0 |
| Naididae spp. | 0 | 0 | 0 | 29 | 0 | 0 | 0 | 0 | 0 | 0 | 0 | 2 | 0 | 0 | 0 | 0 | 0 | 0 | 0 | 0 | 59 | 0 | 0 | 0 | 0 | 0 | 0 | 0 | 0 | 0 | 0 | 0 | 0 | 0 | 1 |
| Oligochaeta spp. | 0 | 2 | 3 | 2 | 1 | 0 | 0 | 0 | 0 | 0 | 0 | 0 | 0 | 1 | 4 | 0 | 1 | 0 | 2 | 11 | 0 | 0 | 0 | 0 | 0 | 13 | 15 | 40 | 56 | 35 | 24 | 1 | 1 | 3 | 0 |
| Tubificoides benedii | 8 | 57 | 92 | 78 | 18 | 183 | 0 | 6 | 43 | 5 | 2 | 0 | 0 | 0 | 0 | 0 | 0 | 0 | 0 | 0 | 0 | 1 | 0 | 0 | 0 | 0 | 0 | 0 | 0 | 0 | 0 | 0 | 0 | 0 | 0 |
| **Mollusca** |  |  |  |  |  |  |  |  |  |  |  |  |  |  |  |  |  |  |  |  |  |  |  |  |  |  |  |  |  |  |  |  |  |  |  |
| Cerastoderma edule | 0 | 1 | 0 | 1 | 0 | 0 | 0 | 0 | 0 | 0 | 0 | 0 | 0 | 0 | 0 | 0 | 0 | 0 | 0 | 0 | 0 | 0 | 0 | 0 | 0 | 0 | 0 | 0 | 0 | 0 | 0 | 0 | 0 | 0 | 0 |
| Cerastoderma glaucum | 0 | 0 | 0 | 0 | 0 | 0 | 0 | 0 | 0 | 0 | 1 | 17 | 8 | 0 | 8 | 0 | 3 | 1 | 4 | 0 | 17 | 4 | 0 | 9 | 30 | 0 | 0 | 0 | 6 | 0 | 0 | 0 | 0 | 0 | 0 |
| Epilepton clarkiae | 0 | 0 | 1 | 0 | 0 | 0 | 0 | 0 | 0 | 0 | 0 | 0 | 0 | 0 | 0 | 0 | 0 | 0 | 0 | 0 | 0 | 0 | 0 | 0 | 0 | 0 | 0 | 0 | 0 | 0 | 0 | 0 | 0 | 0 | 0 |
| Macoma balthica | 4 | 0 | 3 | 3 | 1 | 0 | 1 | 0 | 0 | 0 | 0 | 1 | 0 | 0 | 0 | 2 | 0 | 7 | 2 | 9 | 2 | 5 | 0 | 4 | 2 | 160 | 174 | 139 | 252 | 57 | 6 | 16 | 25 | 11 | 1 |
| Mya arenaria | 1 | 0 | 1 | 0 | 1 | 1 | 1 | 1 | 1 | 0 | 3 | 4 | 4 | 7 | 2 | 17 | 32 | 14 | 20 | 5 | 0 | 0 | 0 | 0 | 2 | 0 | 0 | 0 | 0 | 0 | 0 | 0 | 0 | 0 | 0 |
| Mytilus edulis | 0 | 0 | 0 | 0 | 0 | 0 | 0 | 1 | 0 | 0 | 0 | 0 | 0 | 1 | 0 | 3 | 14 | 0 | 5 | 0 | 0 | 1 | 0 | 0 | 0 | 0 | 0 | 0 | 0 | 0 | 0 | 0 | 0 | 0 | 0 |
| Parvicardium scabrum | 0 | 0 | 0 | 0 | 0 | 2 | 0 | 2 | 0 | 0 | 0 | 0 | 8 | 0 | 0 | 0 | 0 | 0 | 0 | 0 | 0 | 0 | 0 | 0 | 0 | 0 | 0 | 0 | 0 | 0 | 0 | 0 | 0 | 0 | 0 |
| Scrobicularia plana | 0 | 0 | 0 | 0 | 0 | 0 | 0 | 0 | 0 | 0 | 0 | 0 | 0 | 0 | 0 | 0 | 0 | 0 | 0 | 1 | 0 | 0 | 0 | 0 | 0 | 0 | 0 | 0 | 0 | 0 | 0 | 0 | 0 | 0 | 0 |
| Spisula subtruncata | 0 | 0 | 0 | 0 | 0 | 1 | 0 | 0 | 0 | 0 | 0 | 0 | 0 | 0 | 0 | 0 | 0 | 0 | 0 | 0 | 0 | 0 | 0 | 0 | 0 | 0 | 0 | 0 | 0 | 0 | 0 | 0 | 0 | 0 | 0 |
| Polititapes rhomboides | 0 | 0 | 0 | 0 | 0 | 1 | 0 | 2 | 0 | 0 | 0 | 0 | 0 | 0 | 0 | 0 | 0 | 0 | 0 | 0 | 0 | 0 | 0 | 0 | 0 | 0 | 0 | 0 | 0 | 0 | 0 | 0 | 0 | 0 | 0 |
| Bittium reticulatum | 0 | 0 | 0 | 0 | 0 | 0 | 18 | 3 | 3 | 0 | 0 | 0 | 0 | 0 | 0 | 0 | 0 | 0 | 0 | 0 | 0 | 0 | 0 | 0 | 0 | 0 | 0 | 0 | 0 | 0 | 0 | 0 | 0 | 0 | 0 |
| Hydrobia spp. | 4 | 2 | 3 | 2 | 5 | 353 | 311 | 694 | 228 | 3 | 360 | 235 | 265 | 1163 | 393 | 0 | 195 | 121 | 220 | 13 | 0 | 2 | 0 | 0 | 6 | 25 | 8 | 18 | 164 | 62 | 0 | 0 | 0 | 0 | 0 |
| Littorina spp. | 0 | 0 | 0 | 0 | 0 | 0 | 1 | 9 | 2 | 1 | 0 | 0 | 0 | 0 | 0 | 0 | 0 | 0 | 0 | 0 | 0 | 0 | 0 | 0 | 0 | 0 | 0 | 0 | 0 | 0 | 0 | 0 | 0 | 0 | 0 |
| Potamopyrgus antipodarum | 0 | 0 | 0 | 0 | 0 | 0 | 0 | 0 | 0 | 0 | 0 | 0 | 0 | 0 | 0 | 0 | 0 | 0 | 0 | 0 | 0 | 0 | 0 | 0 | 0 | 1 | 1 | 1 | 23 | 7 | 0 | 0 | 1 | 0 | 0 |
| Radix peregra | 0 | 0 | 0 | 0 | 0 | 0 | 0 | 0 | 0 | 0 | 0 | 0 | 0 | 0 | 0 | 0 | 0 | 0 | 0 | 0 | 0 | 0 | 0 | 1 | 0 | 0 | 0 | 0 | 0 | 0 | 0 | 0 | 0 | 0 | 0 |
| Retusa obtusa | 0 | 0 | 0 | 1 | 0 | 0 | 0 | 0 | 0 | 0 | 0 | 0 | 0 | 0 | 0 | 0 | 0 | 0 | 0 | 0 | 0 | 0 | 0 | 0 | 0 | 0 | 0 | 0 | 0 | 0 | 0 | 0 | 0 | 0 | 0 |
| Theodoxus fluviatilis | 0 | 0 | 0 | 0 | 0 | 0 | 0 | 0 | 0 | 0 | 1 | 0 | 0 | 0 | 0 | 0 | 0 | 0 | 0 | 0 | 0 | 0 | 0 | 0 | 0 | 2 | 0 | 0 | 0 | 0 | 0 | 0 | 0 | 0 | 0 |
| Lepidochiton cinereus | 0 | 0 | 0 | 0 | 0 | 0 | 2 | 0 | 0 | 0 | 0 | 0 | 0 | 0 | 0 | 0 | 0 | 0 | 0 | 0 | 0 | 0 | 0 | 0 | 0 | 0 | 0 | 0 | 0 | 0 | 0 | 0 | 0 | 0 | 0 |
| **Nemertea** |  |  |  |  |  |  |  |  |  |  |  |  |  |  |  |  |  |  |  |  |  |  |  |  |  |  |  |  |  |  |  |  |  |  |  |
| Cyanophthalma obscura | 0 | 0 | 0 | 0 | 0 | 0 | 0 | 0 | 0 | 0 | 0 | 0 | 0 | 0 | 0 | 0 | 0 | 0 | 0 | 0 | 0 | 0 | 0 | 0 | 2 | 4 | 1 | 1 | 4 | 2 | 9 | 3 | 3 | 3 | 1 |
| **Arthropoda** |  |  |  |  |  |  |  |  |  |  |  |  |  |  |  |  |  |  |  |  |  |  |  |  |  |  |  |  |  |  |  |  |  |  |  |
| Amphithoe rubicata | 0 | 0 | 0 | 0 | 0 | 0 | 0 | 0 | 0 | 0 | 0 | 0 | 0 | 0 | 0 | 32 | 28 | 0 | 6 | 0 | 0 | 0 | 0 | 0 | 0 | 0 | 0 | 0 | 0 | 0 | 0 | 0 | 0 | 0 | 0 |
| Carcinus maenas | 0 | 0 | 0 | 0 | 1 | 0 | 0 | 4 | 0 | 0 | 0 | 0 | 0 | 0 | 0 | 0 | 0 | 0 | 0 | 0 | 0 | 0 | 0 | 0 | 0 | 0 | 0 | 0 | 0 | 0 | 0 | 0 | 0 | 0 | 0 |
| Corophium multisetosum | 0 | 0 | 0 | 0 | 0 | 0 | 0 | 0 | 0 | 0 | 0 | 0 | 0 | 0 | 0 | 0 | 0 | 0 | 0 | 0 | 0 | 0 | 0 | 0 | 0 | 0 | 0 | 0 | 0 | 0 | 70 | 1 | 0 | 0 | 0 |
| Corophium volutator | 2 | 3 | 8 | 4 | 4 | 0 | 1 | 1 | 0 | 0 | 0 | 0 | 0 | 0 | 0 | 3 | 5 | 0 | 2 | 1 | 0 | 7 | 0 | 14 | 11 | 0 | 0 | 0 | 0 | 44 | 0 | 0 | 0 | 0 | 0 |
| Crangon crangon | 1 | 3 | 0 | 2 | 0 | 0 | 0 | 0 | 0 | 0 | 0 | 0 | 0 | 0 | 0 | 1 | 0 | 0 | 0 | 0 | 0 | 0 | 0 | 0 | 0 | 0 | 0 | 0 | 0 | 0 | 0 | 0 | 0 | 0 | 0 |
| Cyathura carinata | 0 | 0 | 0 | 0 | 0 | 0 | 0 | 0 | 0 | 0 | 6 | 10 | 13 | 8 | 6 | 0 | 1 | 0 | 1 | 0 | 0 | 0 | 0 | 0 | 0 | 0 | 0 | 0 | 0 | 0 | 0 | 0 | 0 | 0 | 0 |
| Gammaridae spp. | 0 | 0 | 0 | 0 | 0 | 0 | 0 | 0 | 0 | 0 | 0 | 0 | 0 | 0 | 0 | 0 | 0 | 0 | 0 | 0 | 0 | 0 | 0 | 3 | 0 | 0 | 0 | 0 | 0 | 0 | 0 | 0 | 0 | 0 | 0 |
| Gammarus duebeni | 0 | 0 | 0 | 0 | 0 | 0 | 0 | 0 | 0 | 0 | 0 | 0 | 0 | 0 | 0 | 0 | 0 | 0 | 0 | 0 | 57 | 42 | 3 | 30 | 0 | 0 | 0 | 0 | 0 | 0 | 0 | 0 | 0 | 0 | 0 |
| Gammarus pulex | 0 | 0 | 0 | 0 | 0 | 0 | 0 | 0 | 0 | 0 | 0 | 0 | 0 | 0 | 0 | 0 | 0 | 0 | 0 | 0 | 0 | 0 | 0 | 0 | 0 | 0 | 0 | 0 | 0 | 0 | 1 | 0 | 0 | 0 | 9 |
| Gammarus salinus | 0 | 0 | 0 | 0 | 0 | 0 | 0 | 0 | 0 | 0 | 0 | 0 | 1 | 0 | 0 | 0 | 0 | 0 | 0 | 0 | 0 | 0 | 0 | 0 | 0 | 0 | 0 | 0 | 0 | 0 | 0 | 0 | 0 | 0 | 1 |
| Heterotanais oerstedi | 0 | 0 | 0 | 0 | 0 | 0 | 0 | 0 | 0 | 0 | 0 | 0 | 0 | 0 | 0 | 0 | 0 | 0 | 1 | 0 | 0 | 0 | 0 | 0 | 0 | 0 | 0 | 0 | 0 | 0 | 0 | 0 | 0 | 0 | 0 |
| Idoitea balthica | 0 | 0 | 0 | 0 | 0 | 0 | 0 | 0 | 0 | 0 | 0 | 0 | 0 | 5 | 0 | 1 | 0 | 0 | 0 | 0 | 0 | 0 | 0 | 0 | 0 | 0 | 0 | 0 | 0 | 0 | 0 | 0 | 0 | 0 | 0 |
| Idoitea chelipes | 0 | 0 | 0 | 0 | 0 | 0 | 0 | 0 | 0 | 0 | 0 | 0 | 4 | 5 | 0 | 0 | 0 | 0 | 0 | 0 | 2 | 0 | 0 | 0 | 0 | 0 | 0 | 0 | 0 | 0 | 0 | 0 | 0 | 0 | 0 |
| Lekanesphaera hookeri | 0 | 0 | 0 | 0 | 0 | 0 | 0 | 0 | 0 | 0 | 2 | 5 | 16 | 11 | 10 | 1 | 0 | 0 | 0 | 0 | 0 | 0 | 0 | 0 | 0 | 0 | 0 | 0 | 0 | 0 | 0 | 0 | 0 | 0 | 0 |
| Microdeutopus gryllotalpa | 0 | 0 | 0 | 0 | 0 | 0 | 0 | 0 | 0 | 0 | 0 | 0 | 0 | 0 | 0 | 0 | 7 | 0 | 0 | 0 | 0 | 0 | 0 | 0 | 0 | 0 | 0 | 0 | 0 | 0 | 0 | 0 | 0 | 0 | 0 |
| Mysida sp. | 0 | 0 | 0 | 1 | 0 | 0 | 0 | 0 | 0 | 0 | 0 | 0 | 0 | 0 | 0 | 0 | 0 | 0 | 0 | 0 | 0 | 0 | 0 | 0 | 0 | 0 | 0 | 1 | 0 | 0 | 1 | 0 | 0 | 0 | 0 |
| Palaemon adspersus | 0 | 0 | 0 | 0 | 0 | 0 | 0 | 0 | 0 | 0 | 0 | 0 | 0 | 0 | 0 | 1 | 0 | 0 | 0 | 0 | 0 | 0 | 0 | 0 | 0 | 0 | 0 | 0 | 0 | 0 | 0 | 0 | 0 | 0 | 0 |
| Tanaidae spp. | 0 | 0 | 0 | 0 | 0 | 0 | 0 | 0 | 0 | 0 | 0 | 0 | 0 | 0 | 0 | 0 | 0 | 0 | 0 | 0 | 1 | 0 | 0 | 0 | 0 | 0 | 0 | 0 | 0 | 0 | 0 | 0 | 0 | 0 | 0 |
| Harpacticoida sp. | 0 | 0 | 0 | 0 | 0 | 0 | 0 | 0 | 0 | 0 | 0 | 0 | 0 | 0 | 0 | 0 | 0 | 0 | 0 | 0 | 0 | 0 | 0 | 0 | 0 | 0 | 0 | 0 | 1 | 0 | 0 | 0 | 0 | 0 | 0 |
| Ostracoda sp. | 0 | 0 | 0 | 0 | 0 | 0 | 0 | 0 | 0 | 0 | 0 | 0 | 0 | 0 | 0 | 0 | 0 | 0 | 0 | 0 | 0 | 0 | 0 | 0 | 0 | 2 | 3 | 5 | 3 | 4 | 0 | 0 | 0 | 0 | 0 |
| Chironomida spp. | 0 | 0 | 0 | 0 | 1 | 0 | 0 | 1 | 0 | 3 | 0 | 0 | 0 | 0 | 0 | 2 | 4 | 0 | 0 | 0 | 5 | 5 | 0 | 11 | 8 | 0 | 2 | 1 | 102 | 6 | 9 | 0 | 0 | 0 | 0 |
| Coleoptera spp. | 0 | 0 | 0 | 0 | 0 | 0 | 0 | 0 | 0 | 0 | 0 | 1 | 8 | 2 | 0 | 0 | 0 | 0 | 0 | 0 | 3 | 1 | 1 | 2 | 0 | 0 | 0 | 0 | 0 | 0 | 0 | 0 | 0 | 0 | 0 |
| Haliplus sp. | 0 | 0 | 0 | 0 | 0 | 0 | 0 | 0 | 0 | 0 | 0 | 0 | 0 | 0 | 0 | 0 | 0 | 0 | 0 | 0 | 0 | 0 | 0 | 0 | 0 | 0 | 0 | 0 | 1 | 0 | 0 | 0 | 0 | 0 | 0 |
| Daphnia sp. | 0 | 0 | 0 | 0 | 0 | 0 | 0 | 0 | 0 | 0 | 0 | 0 | 0 | 0 | 0 | 0 | 0 | 0 | 0 | 0 | 0 | 0 | 0 | 0 | 0 | 0 | 0 | 0 | 3 | 0 | 0 | 0 | 0 | 0 | 0 |
| **Nematoda** |  |  |  |  |  |  |  |  |  |  |  |  |  |  |  |  |  |  |  |  |  |  |  |  |  |  |  |  |  |  |  |  |  |  |  |
| Nematoda spp. | 1 | 2 | 8 | 5 | 0 | 0 | 0 | 0 | 0 | 0 | 0 | 0 | 0 | 1 | 0 | 0 | 0 | 0 | 0 | 0 | 0 | 0 | 0 | 0 | 0 | 0 | 1 | 0 | 1 | 0 | 2 | 3 | 0 | 0 | 0 |

Supplementary table 1B: Results of quantitative species diversity analysis, temporal study. Number of individuals of each species pr. replicate core (706,5 cm^2^). Abbreviations for sample points SA (Saltö August 2018), SB (Saltö November 2018), SC (Saltö April 2019), SD (Saltö August 2019), HA (Herslev August 2018), HB (Herslev November 2018), HC (Herslev April 2019), HD (Herslev August 2019), ÖA (Öland August 2018), ÖB (Öland November 2018), ÖC (Öland April 2019), ÖD (Öland August 2019), each sample point is represented by 5 replicates.

| **Species** | **SA1** | **SA2** | **SA3** | **SA4** | **SA5** | **HA1** | **HA2** | **HA3** | **HA4** | **HA5** | **ÖA1** | **ÖA2** | **ÖA3** | **ÖA4** | **ÖA5** | **SB1** | **SB2** | **SB3** | **SB4** | **SB5** | **HB1** | **HB2** | **HB3** | **HB4** | **HB5** | **ÖB1** | **ÖB2** | **ÖB3** | **ÖB4** | **ÖB5** |
| --- | --- | --- | --- | --- | --- | --- | --- | --- | --- | --- | --- | --- | --- | --- | --- | --- | --- | --- | --- | --- | --- | --- | --- | --- | --- | --- | --- | --- | --- | --- |
| **Annelida** |  |  |  |  |  |  |  |  |  |  |  |  |  |  |  |  |  |  |  |  |  |  |  |  |  |  |  |  |  |  |
| Hediste diversicolor | 33 | 7 | 7 | 2 | 0 | 11 | 31 | 30 | 43 | 14 | 38 | 7 | 22 | 31 | 16 | 0 | 3 | 1 | 3 | 1 | 42 | 36 | 32 | 19 | 9 | 4 | 2 | 4 | 48 | 34 |
| Arenicola marina | 0 | 0 | 0 | 3 | 0 | 0 | 0 | 0 | 0 | 0 | 0 | 0 | 0 | 0 | 0 | 0 | 0 | 4 | 0 | 0 | 0 | 0 | 0 | 0 | 0 | 0 | 0 | 0 | 0 | 0 |
| Nerilla antennata | 0 | 0 | 0 | 0 | 0 | 0 | 0 | 0 | 0 | 0 | 1 | 0 | 0 | 0 | 0 | 0 | 0 | 0 | 0 | 0 | 0 | 0 | 0 | 0 | 0 | 0 | 0 | 0 | 0 | 0 |
| Polydora cornuta | 1 | 0 | 0 | 0 | 0 | 0 | 0 | 0 | 0 | 0 | 0 | 0 | 0 | 0 | 0 | 0 | 2 | 0 | 1 | 1 | 0 | 0 | 2 | 0 | 2 | 0 | 0 | 0 | 0 | 0 |
| Pygospio elegans | 4 | 0 | 0 | 1 | 0 | 0 | 0 | 4 | 5 | 0 | 0 | 0 | 0 | 0 | 0 | 0 | 0 | 0 | 0 | 0 | 2 | 0 | 3 | 0 | 0 | 0 | 0 | 0 | 0 | 0 |
| Heteromastus filiformis | 0 | 0 | 0 | 0 | 4 | 0 | 0 | 0 | 0 | 0 | 0 | 0 | 0 | 0 | 0 | 1 | 6 | 3 | 1 | 3 | 0 | 0 | 0 | 0 | 0 | 0 | 0 | 0 | 0 | 0 |
| Capitella giardi | 0 | 0 | 0 | 0 | 0 | 0 | 0 | 0 | 0 | 0 | 0 | 0 | 0 | 0 | 0 | 1 | 3 | 8 | 2 | 7 | 0 | 0 | 0 | 0 | 0 | 0 | 0 | 0 | 0 | 0 |
| Capitella capitata | 0 | 0 | 0 | 0 | 0 | 0 | 0 | 0 | 0 | 0 | 0 | 0 | 0 | 0 | 0 | 0 | 5 | 3 | 1 | 8 | 0 | 0 | 0 | 0 | 0 | 0 | 0 | 0 | 0 | 0 |
| Mediomastus fragilis | 0 | 0 | 0 | 0 | 1 | 0 | 0 | 0 | 0 | 0 | 0 | 0 | 0 | 0 | 0 | 0 | 1 | 0 | 0 | 0 | 0 | 0 | 0 | 0 | 0 | 0 | 0 | 0 | 0 | 0 |
| Nereis virens | 1 | 0 | 0 | 0 | 0 | 0 | 0 | 0 | 0 | 0 | 0 | 0 | 0 | 0 | 0 | 0 | 1 | 0 | 0 | 1 | 0 | 0 | 0 | 0 | 0 | 0 | 0 | 0 | 0 | 0 |
| Eteone longa | 0 | 0 | 0 | 0 | 0 | 0 | 0 | 0 | 0 | 0 | 0 | 0 | 0 | 0 | 0 | 0 | 0 | 0 | 0 | 0 | 0 | 0 | 0 | 0 | 0 | 0 | 0 | 0 | 0 | 0 |
| Polydora sp. | 0 | 0 | 2 | 0 | 0 | 0 | 0 | 0 | 0 | 0 | 0 | 0 | 0 | 0 | 0 | 0 | 0 | 0 | 0 | 0 | 0 | 0 | 0 | 0 | 0 | 0 | 0 | 0 | 0 | 3 |
| Capitellidae sp. | 0 | 0 | 0 | 0 | 0 | 0 | 0 | 0 | 0 | 0 | 0 | 0 | 0 | 1 | 0 | 0 | 1 | 0 | 0 | 1 | 0 | 0 | 0 | 0 | 0 | 0 | 0 | 0 | 0 | 0 |
| Polydora ciliata | 0 | 0 | 0 | 0 | 0 | 0 | 0 | 0 | 0 | 0 | 0 | 0 | 0 | 0 | 0 | 0 | 0 | 0 | 0 | 4 | 1 | 0 | 1 | 1 | 0 | 0 | 0 | 0 | 0 | 0 |
| Eteone sp. | 0 | 0 | 0 | 0 | 0 | 0 | 0 | 0 | 0 | 0 | 0 | 0 | 0 | 0 | 0 | 0 | 0 | 0 | 0 | 0 | 0 | 0 | 0 | 0 | 0 | 0 | 0 | 0 | 0 | 0 |
| Syllidia armata | 0 | 0 | 0 | 0 | 0 | 0 | 0 | 0 | 0 | 0 | 0 | 0 | 0 | 0 | 0 | 0 | 1 | 0 | 0 | 0 | 0 | 0 | 0 | 0 | 0 | 0 | 0 | 0 | 0 | 0 |
| Scoloplos armiger | 0 | 0 | 0 | 0 | 0 | 0 | 0 | 0 | 0 | 0 | 0 | 0 | 0 | 0 | 0 | 0 | 0 | 0 | 0 | 1 | 0 | 0 | 0 | 0 | 0 | 0 | 0 | 0 | 0 | 0 |
| Spionidae sp | 0 | 0 | 0 | 0 | 0 | 0 | 0 | 0 | 0 | 0 | 0 | 0 | 0 | 0 | 0 | 0 | 0 | 0 | 0 | 1 | 1 | 0 | 1 | 0 | 0 | 0 | 0 | 0 | 0 | 0 |
| Marenzelleria sp. | 0 | 0 | 0 | 1 | 0 | 0 | 0 | 0 | 0 | 0 | 0 | 1 | 1 | 2 | 1 | 0 | 0 | 0 | 0 | 0 | 0 | 0 | 0 | 0 | 0 | 1 | 3 | 1 | 1 | 1 |
| Spio sp. | 0 | 0 | 0 | 0 | 0 | 0 | 0 | 0 | 0 | 0 | 0 | 0 | 0 | 0 | 0 | 0 | 0 | 0 | 0 | 0 | 0 | 0 | 0 | 0 | 0 | 0 | 0 | 0 | 0 | 0 |
| Tubificoides benedii | 183 | 0 | 6 | 43 | 5 | 2 | 0 | 0 | 0 | 0 | 0 | 1 | 0 | 0 | 0 | 16 | 26 | 8 | 14 | 11 | 0 | 0 | 0 | 0 | 0 | 0 | 0 | 0 | 0 | 0 |
| Enchytraeidae sp. | 0 | 0 | 0 | 0 | 0 | 0 | 14 | 0 | 0 | 0 | 0 | 1 | 0 | 0 | 0 | 0 | 0 | 0 | 0 | 0 | 0 | 0 | 0 | 0 | 0 | 0 | 0 | 0 | 0 | 0 |
| Tubificidae spp. | 0 | 0 | 0 | 0 | 0 | 0 | 2 | 0 | 0 | 0 | 59 | 0 | 0 | 0 | 0 | 0 | 0 | 0 | 0 | 0 | 0 | 0 | 0 | 0 | 0 | 0 | 0 | 0 | 0 | 0 |
| Oligochaeta spp. | 0 | 0 | 0 | 0 | 0 | 0 | 0 | 0 | 1 | 4 | 0 | 0 | 0 | 0 | 0 | 2 | 2 | 1 | 0 | 4 | 0 | 0 | 0 | 2 | 0 | 14 | 4 | 19 | 31 | 52 |
| **Mollusca** |  |  |  |  |  |  |  |  |  |  |  |  |  |  |  |  |  |  |  |  |  |  |  |  |  |  |  |  |  |  |
| Macoma balthica | 0 | 1 | 0 | 0 | 0 | 0 | 1 | 0 | 0 | 0 | 2 | 5 | 0 | 4 | 2 | 0 | 0 | 0 | 0 | 1 | 0 | 0 | 0 | 0 | 0 | 0 | 1 | 0 | 5 | 6 |
| Mytilus edulis | 0 | 0 | 1 | 0 | 0 | 0 | 0 | 0 | 1 | 0 | 0 | 1 | 0 | 0 | 0 | 0 | 0 | 0 | 0 | 0 | 0 | 0 | 0 | 0 | 0 | 0 | 0 | 0 | 0 | 0 |
| Spisula subtruncata | 1 | 0 | 0 | 0 | 0 | 0 | 0 | 0 | 0 | 0 | 0 | 0 | 0 | 0 | 0 | 0 | 0 | 0 | 0 | 0 | 0 | 0 | 0 | 0 | 0 | 0 | 0 | 0 | 0 | 0 |
| Polititapes rhomboides | 1 | 0 | 2 | 0 | 0 | 0 | 0 | 0 | 0 | 0 | 0 | 0 | 0 | 0 | 0 | 0 | 0 | 0 | 0 | 0 | 0 | 0 | 0 | 0 | 0 | 0 | 0 | 0 | 0 | 0 |
| Parvicardium scabrum | 2 | 0 | 2 | 0 | 0 | 0 | 0 | 0 | 0 | 0 | 0 | 0 | 0 | 0 | 0 | 0 | 0 | 0 | 0 | 0 | 0 | 0 | 0 | 0 | 0 | 0 | 0 | 0 | 0 | 0 |
| Cerastoderma edule | 0 | 0 | 0 | 0 | 0 | 0 | 0 | 0 | 0 | 0 | 0 | 0 | 0 | 0 | 0 | 0 | 0 | 0 | 0 | 0 | 0 | 0 | 0 | 0 | 0 | 0 | 0 | 0 | 0 | 0 |
| Cerastoderma glaucum | 0 | 0 | 0 | 0 | 0 | 1 | 17 | 8 | 0 | 8 | 17 | 4 | 0 | 9 | 30 | 0 | 0 | 0 | 0 | 0 | 1 | 0 | 3 | 1 | 0 | 7 | 0 | 1 | 37 | 53 |
| Scrobicularia plana | 0 | 0 | 0 | 0 | 0 | 0 | 0 | 0 | 0 | 0 | 0 | 0 | 0 | 0 | 0 | 0 | 0 | 1 | 0 | 0 | 0 | 0 | 0 | 0 | 0 | 0 | 0 | 0 | 0 | 0 |
| Abra tenius | 0 | 0 | 0 | 0 | 0 | 0 | 0 | 0 | 0 | 0 | 0 | 0 | 0 | 0 | 0 | 0 | 0 | 0 | 0 | 0 | 0 | 0 | 0 | 0 | 0 | 0 | 0 | 0 | 0 | 0 |
| Crassostrea gigas | 0 | 0 | 0 | 0 | 0 | 0 | 0 | 0 | 0 | 0 | 0 | 0 | 0 | 0 | 0 | 0 | 0 | 0 | 0 | 0 | 0 | 0 | 0 | 0 | 0 | 0 | 0 | 0 | 0 | 0 |
| Modiolula phaseolina | 0 | 0 | 0 | 0 | 0 | 0 | 0 | 0 | 0 | 0 | 0 | 0 | 0 | 0 | 0 | 0 | 0 | 0 | 0 | 0 | 0 | 0 | 0 | 0 | 0 | 0 | 0 | 0 | 0 | 0 |
| Mya arenaria | 1 | 1 | 1 | 1 | 0 | 3 | 4 | 4 | 7 | 2 | 0 | 0 | 0 | 0 | 2 | 0 | 1 | 0 | 0 | 1 | 5 | 2 | 2 | 0 | 2 | 0 | 0 | 0 | 6 | 7 |
| Parvicardium ovale | 0 | 0 | 0 | 0 | 0 | 0 | 0 | 0 | 0 | 0 | 0 | 0 | 0 | 0 | 0 | 0 | 0 | 0 | 0 | 0 | 0 | 0 | 0 | 0 | 0 | 0 | 0 | 0 | 0 | 0 |
| Hydrobia spp. | 353 | 311 | 694 | 228 | 3 | 360 | 235 | 265 | 1163 | 393 | 0 | 2 | 0 | 0 | 6 | 5 | 183 | 12 | 22 | 22 | 778 | 773 | 664 | 453 | 272 | 0 | 2 | 2 | 35 | 19 |
| Theodoxus fluviatilis | 0 | 0 | 0 | 0 | 0 | 1 | 0 | 0 | 0 | 0 | 0 | 0 | 0 | 0 | 0 | 0 | 0 | 0 | 0 | 0 | 0 | 0 | 4 | 0 | 1 | 0 | 0 | 0 | 0 | 0 |
| Bittium reticulatum | 0 | 18 | 3 | 3 | 0 | 0 | 0 | 0 | 0 | 0 | 0 | 0 | 0 | 0 | 0 | 1 | 2 | 2 | 3 | 8 | 0 | 0 | 0 | 0 | 0 | 0 | 0 | 0 | 0 | 0 |
| Nassarius reticulatus | 0 | 0 | 0 | 0 | 0 | 0 | 0 | 0 | 0 | 0 | 0 | 0 | 0 | 0 | 0 | 0 | 0 | 0 | 1 | 2 | 0 | 0 | 0 | 0 | 0 | 0 | 0 | 0 | 0 | 0 |
| Radix peregra | 0 | 0 | 0 | 0 | 0 | 0 | 0 | 0 | 0 | 0 | 0 | 0 | 0 | 1 | 0 | 0 | 0 | 0 | 0 | 0 | 0 | 0 | 0 | 0 | 0 | 0 | 0 | 0 | 0 | 0 |
| Littorina spp. | 0 | 1 | 9 | 2 | 1 | 0 | 0 | 0 | 0 | 0 | 0 | 0 | 0 | 0 | 0 | 0 | 1 | 0 | 0 | 1 | 0 | 2 | 0 | 0 | 0 | 0 | 0 | 0 | 0 | 0 |
| Pusillina sarsi | 0 | 0 | 0 | 0 | 0 | 0 | 0 | 0 | 0 | 0 | 0 | 0 | 0 | 0 | 0 | 0 | 0 | 0 | 0 | 1 | 0 | 0 | 0 | 0 | 0 | 0 | 0 | 0 | 0 | 0 |
| Retusa umbillicata | 0 | 0 | 0 | 0 | 0 | 0 | 0 | 0 | 0 | 0 | 0 | 0 | 0 | 0 | 0 | 0 | 0 | 0 | 0 | 0 | 0 | 0 | 0 | 0 | 0 | 0 | 0 | 0 | 0 | 0 |
| Retusa truncatula | 0 | 0 | 0 | 0 | 0 | 0 | 0 | 0 | 0 | 0 | 0 | 0 | 0 | 0 | 0 | 0 | 0 | 0 | 0 | 0 | 0 | 0 | 0 | 0 | 0 | 0 | 0 | 0 | 0 | 0 |
| Majidae sp | 0 | 0 | 0 | 0 | 0 | 0 | 0 | 0 | 0 | 0 | 0 | 0 | 0 | 0 | 0 | 0 | 0 | 0 | 0 | 0 | 0 | 0 | 0 | 0 | 0 | 0 | 0 | 0 | 0 | 0 |
| Elysia viridis | 0 | 0 | 0 | 0 | 0 | 0 | 0 | 0 | 0 | 0 | 0 | 0 | 0 | 0 | 0 | 0 | 0 | 0 | 0 | 0 | 0 | 0 | 0 | 0 | 0 | 0 | 0 | 0 | 0 | 0 |
| Lepidochiton cinereus | 0 | 2 | 0 | 0 | 0 | 0 | 0 | 0 | 0 | 0 | 0 | 0 | 0 | 0 | 0 | 0 | 0 | 0 | 0 | 0 | 0 | 0 | 0 | 0 | 0 | 0 | 0 | 0 | 0 | 0 |
| **Nemertea** |  |  |  |  |  |  |  |  |  |  |  |  |  |  |  |  |  |  |  |  |  |  |  |  |  |  |  |  |  |  |
| Cyanophthalma obscura | 0 | 0 | 0 | 0 | 0 | 0 | 0 | 0 | 0 | 0 | 0 | 0 | 0 | 0 | 2 | 0 | 0 | 0 | 0 | 0 | 0 | 0 | 0 | 0 | 0 | 0 | 0 | 0 | 0 | 0 |
| **Arthropoda** |  |  |  |  |  |  |  |  |  |  |  |  |  |  |  |  |  |  |  |  |  |  |  |  |  |  |  |  |  |  |
| Cyathura carinata | 0 | 0 | 0 | 0 | 0 | 6 | 10 | 13 | 8 | 6 | 0 | 0 | 0 | 0 | 0 | 0 | 0 | 0 | 0 | 0 | 11 | 5 | 8 | 14 | 8 | 0 | 0 | 0 | 0 | 0 |
| Lekanesphaera hookeri | 0 | 0 | 0 | 0 | 0 | 2 | 5 | 16 | 7 | 0 | 0 | 0 | 0 | 0 | 0 | 0 | 0 | 0 | 0 | 0 | 12 | 20 | 18 | 0 | 2 | 0 | 0 | 0 | 0 | 0 |
| Corophium volutator | 0 | 1 | 1 | 0 | 0 | 0 | 0 | 0 | 0 | 0 | 0 | 7 | 0 | 14 | 11 | 0 | 0 | 0 | 0 | 2 | 0 | 3 | 0 | 0 | 0 | 86 | 10 | 13 | 43 | 36 |
| Amphithoe rubricata | 0 | 0 | 0 | 0 | 0 | 0 | 0 | 0 | 0 | 0 | 0 | 0 | 0 | 0 | 0 | 0 | 1 | 0 | 0 | 0 | 0 | 0 | 0 | 0 | 0 | 0 | 0 | 0 | 0 | 0 |
| Tanaidae spp. | 0 | 0 | 0 | 0 | 0 | 0 | 0 | 0 | 0 | 0 | 1 | 0 | 0 | 0 | 0 | 0 | 0 | 0 | 0 | 0 | 0 | 0 | 0 | 0 | 0 | 0 | 0 | 0 | 0 | 0 |
| Idoitea chelipes | 0 | 0 | 0 | 0 | 0 | 0 | 0 | 4 | 5 | 0 | 2 | 0 | 0 | 0 | 0 | 0 | 0 | 0 | 0 | 0 | 1 | 0 | 0 | 0 | 0 | 2 | 2 | 0 | 1 | 0 |
| Gammarus salinus | 0 | 0 | 0 | 0 | 0 | 0 | 0 | 1 | 0 | 0 | 0 | 0 | 0 | 0 | 0 | 0 | 0 | 0 | 0 | 0 | 0 | 0 | 0 | 0 | 0 | 0 | 0 | 0 | 0 | 0 |
| Microdeutopus gryllotalpa | 0 | 0 | 0 | 0 | 0 | 0 | 0 | 0 | 0 | 0 | 0 | 0 | 0 | 0 | 0 | 0 | 0 | 0 | 0 | 0 | 0 | 0 | 0 | 0 | 0 | 0 | 0 | 0 | 0 | 0 |
| Carcinus maenas | 0 | 0 | 4 | 0 | 0 | 0 | 0 | 0 | 0 | 0 | 0 | 0 | 0 | 0 | 0 | 0 | 0 | 1 | 1 | 0 | 0 | 0 | 0 | 0 | 0 | 0 | 0 | 0 | 0 | 0 |
| Tanais dulongii | 0 | 0 | 0 | 0 | 0 | 0 | 0 | 0 | 0 | 0 | 0 | 0 | 0 | 0 | 0 | 0 | 0 | 0 | 0 | 0 | 0 | 0 | 0 | 0 | 0 | 0 | 0 | 0 | 0 | 0 |
| Gammaridae spp. | 0 | 0 | 0 | 0 | 0 | 0 | 0 | 0 | 0 | 0 | 0 | 0 | 0 | 3 | 0 | 0 | 0 | 0 | 1 | 0 | 0 | 0 | 0 | 0 | 0 | 0 | 0 | 0 | 0 | 0 |
| Gammarus duebeni | 0 | 0 | 0 | 0 | 0 | 0 | 0 | 0 | 0 | 0 | 57 | 42 | 3 | 30 | 0 | 0 | 0 | 0 | 0 | 0 | 0 | 0 | 0 | 0 | 0 | 5 | 3 | 7 | 15 | 0 |
| Palaemon adspersus | 0 | 0 | 0 | 0 | 0 | 0 | 0 | 0 | 0 | 0 | 0 | 0 | 0 | 0 | 0 | 0 | 0 | 0 | 0 | 0 | 0 | 0 | 0 | 0 | 0 | 0 | 0 | 0 | 0 | 0 |
| Mysida sp. | 0 | 0 | 0 | 0 | 0 | 0 | 0 | 0 | 0 | 0 | 0 | 0 | 0 | 0 | 0 | 0 | 0 | 0 | 0 | 0 | 0 | 0 | 0 | 0 | 0 | 0 | 0 | 0 | 0 | 0 |
| Erichthonius difformis | 0 | 0 | 0 | 0 | 0 | 0 | 0 | 0 | 0 | 0 | 0 | 0 | 0 | 0 | 0 | 0 | 0 | 0 | 0 | 0 | 0 | 0 | 0 | 0 | 0 | 0 | 0 | 0 | 0 | 0 |
| Chironomida spp. | 0 | 0 | 1 | 0 | 3 | 0 | 0 | 0 | 0 | 0 | 5 | 5 | 0 | 11 | 8 | 0 | 7 | 7 | 9 | 6 | 0 | 0 | 4 | 0 | 0 | 0 | 0 | 0 | 8 | 13 |
| Coleoptera spp. | 0 | 0 | 0 | 0 | 0 | 0 | 1 | 8 | 2 | 0 | 3 | 1 | 1 | 2 | 0 | 0 | 0 | 0 | 0 | 0 | 2 | 0 | 0 | 1 | 0 | 1 | 0 | 0 | 0 | 0 |
| Anurida maritima | 0 | 0 | 0 | 0 | 0 | 0 | 0 | 0 | 0 | 0 | 0 | 0 | 0 | 0 | 0 | 0 | 0 | 0 | 0 | 0 | 0 | 0 | 0 | 0 | 0 | 0 | 0 | 0 | 0 | 0 |
| **Nematoda** |  |  |  |  |  |  |  |  |  |  |  |  |  |  |  |  |  |  |  |  |  |  |  |  |  |  |  |  |  |  |
| Nematoda spp. | 0 | 0 | 0 | 0 | 0 | 0 | 0 | 0 | 1 | 0 | 0 | 0 | 0 | 0 | 0 | 1 | 2 | 0 | 0 | 2 | 0 | 0 | 0 | 0 | 0 | 0 | 0 | 0 | 0 | 1 |

| **Species** | **SC1** | **SC2** | **SC3** | **SC4** | **SC5** | **HC1** | **HC2** | **HC3** | **HC4** | **HC5** | **ÖC1** | **ÖC2** | **ÖC3** | **ÖC4** | **ÖC5** | **SD1** | **SD2** | **SD3** | **SD4** | **SD5** | **HD1** | **HD2** | **HD3** | **HD4** | **HD5** | **ÖD1** | **ÖD2** | **Öl3** | **ÖD4** | **ÖD5** |
| --- | --- | --- | --- | --- | --- | --- | --- | --- | --- | --- | --- | --- | --- | --- | --- | --- | --- | --- | --- | --- | --- | --- | --- | --- | --- | --- | --- | --- | --- | --- |
| **Annelida** |  |  |  |  |  |  |  |  |  |  |  |  |  |  |  |  |  |  |  |  |  |  |  |  |  |  |  |  |  |  |
| Hediste diversicolor | 3 | 3 | 0 | 0 | 1 | 16 | 37 | 12 | 16 | 14 | 18 | 14 | 12 | 17 | 41 | 16 | 12 | 13 | 11 | 1 | 7 | 15 | 27 | 13 | 20 | 30 | 14 | 35 | 40 | 30 |
| Arenicola marina | 0 | 0 | 1 | 0 | 0 | 0 | 0 | 0 | 1 | 1 | 0 | 0 | 0 | 0 | 0 | 0 | 0 | 0 | 0 | 0 | 1 | 0 | 0 | 0 | 0 | 0 | 0 | 0 | 0 | 0 |
| Nerilla antennata | 0 | 0 | 0 | 0 | 0 | 0 | 0 | 0 | 0 | 0 | 0 | 0 | 0 | 0 | 0 | 0 | 0 | 0 | 0 | 0 | 0 | 0 | 0 | 0 | 0 | 0 | 0 | 0 | 0 | 0 |
| Polydora cornuta | 2 | 4 | 0 | 3 | 1 | 0 | 1 | 0 | 0 | 0 | 0 | 0 | 0 | 0 | 0 | 3 | 6 | 7 | 4 | 0 | 0 | 1 | 1 | 0 | 0 | 0 | 0 | 0 | 0 | 0 |
| Pygospio elegans | 16 | 87 | 24 | 64 | 19 | 0 | 9 | 15 | 5 | 0 | 0 | 0 | 0 | 0 | 4 | 3 | 11 | 28 | 13 | 0 | 0 | 2 | 32 | 5 | 12 | 3 | 1 | 1 | 8 | 13 |
| Heteromastus filiformis | 0 | 0 | 3 | 2 | 6 | 0 | 0 | 0 | 0 | 0 | 0 | 0 | 0 | 0 | 0 | 7 | 14 | 25 | 20 | 11 | 0 | 0 | 0 | 0 | 0 | 0 | 0 | 0 | 0 | 0 |
| Capitella giardi | 0 | 0 | 0 | 9 | 1 | 0 | 0 | 0 | 0 | 0 | 0 | 0 | 0 | 0 | 0 | 6 | 3 | 2 | 4 | 21 | 0 | 0 | 0 | 0 | 0 | 0 | 0 | 0 | 0 | 0 |
| Capitella capitata | 8 | 18 | 15 | 17 | 33 | 0 | 0 | 0 | 0 | 0 | 0 | 0 | 0 | 0 | 0 | 1 | 15 | 19 | 3 | 2 | 0 | 0 | 0 | 0 | 0 | 0 | 0 | 0 | 0 | 0 |
| Mediomastus fragilis | 7 | 3 | 6 | 4 | 11 | 0 | 0 | 0 | 0 | 0 | 0 | 0 | 0 | 0 | 0 | 1 | 5 | 6 | 11 | 16 | 0 | 0 | 0 | 0 | 0 | 0 | 0 | 0 | 0 | 0 |
| Nereis virens | 0 | 0 | 0 | 0 | 0 | 0 | 0 | 0 | 0 | 0 | 0 | 0 | 0 | 0 | 0 | 0 | 0 | 0 | 0 | 0 | 0 | 0 | 0 | 0 | 0 | 0 | 0 | 0 | 0 | 0 |
| Eteone longa | 0 | 2 | 0 | 0 | 0 | 0 | 0 | 0 | 0 | 0 | 0 | 0 | 0 | 0 | 0 | 0 | 0 | 0 | 0 | 0 | 0 | 0 | 0 | 0 | 0 | 0 | 0 | 0 | 0 | 0 |
| Polydora sp. | 0 | 1 | 1 | 1 | 1 | 0 | 0 | 1 | 0 | 0 | 0 | 0 | 0 | 0 | 2 | 3 | 3 | 0 | 0 | 0 | 0 | 0 | 1 | 0 | 0 | 0 | 0 | 0 | 0 | 0 |
| Capitellidae sp. | 0 | 1 | 7 | 0 | 5 | 0 | 0 | 0 | 0 | 0 | 0 | 0 | 0 | 0 | 0 | 3 | 10 | 14 | 4 | 3 | 0 | 0 | 0 | 0 | 0 | 0 | 0 | 0 | 0 | 0 |
| Polydora ciliata | 0 | 0 | 0 | 1 | 0 | 0 | 0 | 0 | 0 | 0 | 0 | 0 | 0 | 0 | 0 | 0 | 3 | 0 | 0 | 0 | 0 | 0 | 0 | 0 | 0 | 0 | 0 | 0 | 0 | 0 |
| Eteone sp. | 0 | 2 | 0 | 0 | 0 | 0 | 0 | 0 | 0 | 0 | 0 | 0 | 0 | 0 | 0 | 0 | 0 | 2 | 0 | 0 | 0 | 0 | 0 | 0 | 0 | 0 | 1 | 0 | 0 | 0 |
| Syllidia armata | 0 | 0 | 0 | 0 | 0 | 0 | 0 | 0 | 0 | 0 | 0 | 0 | 0 | 0 | 0 | 0 | 0 | 0 | 0 | 0 | 0 | 0 | 0 | 0 | 0 | 0 | 0 | 0 | 0 | 0 |
| Scoloplos armiger | 0 | 0 | 0 | 0 | 0 | 0 | 0 | 0 | 0 | 0 | 0 | 0 | 0 | 0 | 0 | 0 | 0 | 0 | 0 | 0 | 0 | 0 | 0 | 0 | 0 | 0 | 0 | 0 | 0 | 0 |
| Spionidae sp | 0 | 0 | 0 | 0 | 0 | 0 | 0 | 0 | 0 | 0 | 0 | 0 | 0 | 0 | 0 | 0 | 1 | 1 | 0 | 0 | 0 | 0 | 0 | 0 | 0 | 0 | 0 | 0 | 0 | 0 |
| Marenzelleria sp. | 0 | 2 | 0 | 0 | 1 | 0 | 0 | 0 | 0 | 0 | 4 | 3 | 2 | 2 | 0 | 0 | 0 | 2 | 0 | 0 | 0 | 0 | 0 | 0 | 0 | 1 | 3 | 1 | 6 | 1 |
| Spio sp. | 0 | 0 | 0 | 2 | 0 | 0 | 0 | 0 | 0 | 0 | 0 | 0 | 0 | 0 | 0 | 0 | 0 | 0 | 0 | 0 | 0 | 0 | 0 | 0 | 0 | 0 | 0 | 0 | 0 | 0 |
| Tubificoides benedii | 32 | 157 | 11 | 108 | 112 | 0 | 0 | 2 | 6 | 0 | 0 | 0 | 0 | 0 | 0 | 5 | 17 | 16 | 7 | 9 | 0 | 0 | 0 | 0 | 0 | 0 | 0 | 0 | 0 | 0 |
| Enchytraeidae sp. | 0 | 0 | 0 | 0 | 0 | 0 | 0 | 0 | 0 | 0 | 0 | 0 | 0 | 0 | 0 | 0 | 0 | 0 | 0 | 0 | 0 | 0 | 0 | 0 | 0 | 0 | 0 | 0 | 0 | 0 |
| Tubificidae spp. | 0 | 0 | 0 | 1 | 0 | 0 | 0 | 0 | 0 | 0 | 0 | 0 | 0 | 0 | 0 | 0 | 0 | 0 | 0 | 0 | 0 | 0 | 0 | 0 | 0 | 0 | 0 | 0 | 0 | 0 |
| Oligochaeta spp. | 0 | 1 | 1 | 0 | 5 | 0 | 0 | 0 | 1 | 0 | 10 | 1 | 10 | 9 | 5 | 1 | 0 | 1 | 2 | 2 | 0 | 0 | 1 | 1 | 0 | 11 | 13 | 31 | 35 | 23 |
| **Mollusca** |  |  |  |  |  |  |  |  |  |  |  |  |  |  |  |  |  |  |  |  |  |  |  |  |  |  |  |  |  |  |
| Macoma balthica | 0 | 1 | 0 | 1 | 1 | 0 | 0 | 0 | 1 | 0 | 3 | 0 | 0 | 2 | 4 | 0 | 2 | 3 | 3 | 1 | 0 | 0 | 0 | 0 | 0 | 6 | 1 | 1 | 1 | 5 |
| Mytilus edulis | 0 | 0 | 0 | 0 | 0 | 0 | 0 | 0 | 0 | 0 | 0 | 0 | 0 | 1 | 0 | 2 | 1 | 1 | 0 | 0 | 0 | 1 | 2 | 0 | 2 | 0 | 0 | 0 | 0 | 0 |
| Spisula subtruncata | 0 | 0 | 0 | 0 | 0 | 0 | 0 | 0 | 0 | 0 | 0 | 0 | 0 | 0 | 0 | 0 | 0 | 0 | 0 | 0 | 0 | 0 | 0 | 0 | 0 | 0 | 0 | 0 | 0 | 0 |
| Polititapes rhomboides | 0 | 0 | 0 | 0 | 0 | 0 | 0 | 0 | 0 | 0 | 0 | 0 | 0 | 0 | 0 | 0 | 3 | 0 | 0 | 0 | 0 | 0 | 0 | 0 | 0 | 0 | 0 | 0 | 0 | 0 |
| Parvicardium scabrum | 0 | 0 | 0 | 0 | 0 | 0 | 0 | 0 | 0 | 0 | 0 | 0 | 0 | 0 | 0 | 0 | 0 | 0 | 0 | 0 | 0 | 0 | 0 | 0 | 0 | 0 | 0 | 0 | 0 | 0 |
| Cerastoderma edule | 1 | 3 | 0 | 0 | 0 | 0 | 0 | 0 | 0 | 0 | 0 | 0 | 0 | 0 | 0 | 4 | 3 | 1 | 4 | 4 | 0 | 0 | 0 | 0 | 0 | 0 | 0 | 0 | 0 | 0 |
| Cerastoderma glaucum | 0 | 0 | 0 | 0 | 0 | 0 | 0 | 0 | 1 | 0 | 4 | 0 | 1 | 1 | 12 | 0 | 0 | 0 | 0 | 0 | 1 | 0 | 7 | 14 | 14 | 6 | 0 | 4 | 6 | 4 |
| Scrobicularia plana | 0 | 0 | 0 | 0 | 0 | 0 | 0 | 0 | 0 | 0 | 0 | 0 | 0 | 0 | 0 | 0 | 0 | 0 | 0 | 0 | 0 | 0 | 0 | 0 | 0 | 0 | 0 | 0 | 0 | 0 |
| Abra tenius | 0 | 0 | 0 | 0 | 0 | 0 | 0 | 0 | 0 | 0 | 0 | 0 | 0 | 0 | 0 | 2 | 1 | 3 | 0 | 0 | 0 | 0 | 0 | 0 | 0 | 0 | 0 | 0 | 0 | 0 |
| Crassostrea gigas | 0 | 0 | 0 | 0 | 0 | 0 | 0 | 0 | 0 | 0 | 0 | 0 | 0 | 0 | 0 | 0 | 1 | 0 | 0 | 0 | 0 | 0 | 0 | 0 | 0 | 0 | 0 | 0 | 0 | 0 |
| Modiolula phaseolina | 0 | 0 | 0 | 0 | 0 | 0 | 0 | 0 | 0 | 0 | 0 | 0 | 0 | 0 | 0 | 2 | 6 | 6 | 19 | 0 | 0 | 0 | 0 | 0 | 0 | 0 | 0 | 0 | 0 | 0 |
| Mya arenaria | 3 | 5 | 1 | 3 | 0 | 4 | 2 | 4 | 5 | 2 | 0 | 0 | 0 | 1 | 5 | 2 | 5 | 4 | 0 | 0 | 1 | 1 | 2 | 2 | 5 | 1 | 0 | 1 | 1 | 1 |
| Parvicardium ovale | 0 | 0 | 0 | 0 | 0 | 0 | 0 | 0 | 0 | 0 | 0 | 0 | 0 | 0 | 0 | 2 | 1 | 3 | 0 | 0 | 0 | 0 | 0 | 0 | 0 | 0 | 0 | 0 | 0 | 0 |
| Hydrobia spp. | 31 | 28 | 22 | 59 | 23 | 245 | 206 | 936 | 332 | 267 | 1 | 1 | 1 | 9 | 20 | 144 | 93 | 77 | 417 | 111 | 504 | 584 | 285 | 336 | 1325 | 8 | 2 | 1 | 8 | 1 |
| Theodoxus fluviatilis | 0 | 0 | 0 | 0 | 0 | 0 | 0 | 1 | 0 | 0 | 0 | 0 | 0 | 0 | 0 | 0 | 0 | 0 | 0 | 0 | 2 | 3 | 2 | 2 | 1 | 0 | 0 | 0 | 0 | 0 |
| Bittium reticulatum | 7 | 15 | 9 | 16 | 12 | 0 | 0 | 0 | 0 | 0 | 0 | 0 | 0 | 0 | 0 | 10 | 5 | 0 | 9 | 0 | 0 | 0 | 0 | 0 | 0 | 0 | 0 | 0 | 0 | 0 |
| Nassarius reticulatus | 0 | 0 | 0 | 0 | 0 | 0 | 0 | 0 | 0 | 0 | 0 | 0 | 0 | 0 | 0 | 0 | 0 | 0 | 0 | 0 | 0 | 0 | 0 | 0 | 0 | 0 | 0 | 0 | 0 | 0 |
| Radix peregra | 0 | 0 | 0 | 0 | 0 | 0 | 0 | 0 | 0 | 0 | 0 | 0 | 0 | 0 | 0 | 0 | 0 | 0 | 0 | 0 | 0 | 0 | 0 | 0 | 0 | 0 | 0 | 0 | 0 | 0 |
| Littorina spp. | 1 | 0 | 0 | 4 | 0 | 0 | 0 | 0 | 0 | 0 | 0 | 0 | 0 | 0 | 0 | 0 | 3 | 0 | 0 | 0 | 4 | 0 | 3 | 7 | 5 | 0 | 0 | 0 | 0 | 0 |
| Pusillina sarsi | 0 | 0 | 0 | 0 | 0 | 0 | 0 | 0 | 0 | 0 | 0 | 0 | 0 | 0 | 0 | 1 | 0 | 31 | 21 | 0 | 0 | 0 | 0 | 0 | 0 | 0 | 0 | 0 | 0 | 0 |
| Retusa umbillicata | 0 | 0 | 0 | 0 | 0 | 0 | 0 | 0 | 0 | 0 | 0 | 0 | 0 | 0 | 0 | 1 | 3 | 2 | 0 | 2 | 0 | 0 | 0 | 0 | 0 | 0 | 0 | 0 | 0 | 0 |
| Retusa truncatula | 0 | 0 | 0 | 0 | 0 | 0 | 0 | 0 | 0 | 0 | 0 | 0 | 0 | 0 | 0 | 0 | 0 | 0 | 4 | 0 | 0 | 0 | 0 | 0 | 0 | 0 | 0 | 0 | 0 | 0 |
| Majidae sp | 0 | 0 | 0 | 0 | 0 | 0 | 0 | 0 | 0 | 0 | 0 | 0 | 0 | 0 | 0 | 0 | 0 | 0 | 1 | 0 | 0 | 0 | 0 | 0 | 0 | 0 | 0 | 0 | 0 | 0 |
| Elysia viridis | 0 | 0 | 0 | 0 | 0 | 0 | 0 | 0 | 0 | 0 | 0 | 0 | 0 | 0 | 0 | 0 | 0 | 0 | 0 | 0 | 0 | 0 | 0 | 0 | 0 | 0 | 0 | 0 | 0 | 0 |
| Lepidochiton cinereus | 0 | 0 | 0 | 0 | 0 | 0 | 0 | 0 | 0 | 0 | 0 | 0 | 0 | 0 | 0 | 1 | 3 | 0 | 0 | 0 | 0 | 0 | 0 | 0 | 0 | 0 | 0 | 0 | 0 | 0 |
| **Nemertea** |  |  |  |  |  |  |  |  |  |  |  |  |  |  |  |  |  |  |  |  |  |  |  |  |  |  |  |  |  |  |
| Cyanophthalma obscura | 0 | 0 | 0 | 0 | 0 | 0 | 0 | 0 | 0 | 0 | 0 | 0 | 0 | 0 | 0 | 0 | 0 | 0 | 0 | 0 | 0 | 0 | 0 | 0 | 0 | 3 | 0 | 0 | 0 | 0 |
| **Arthropoda** |  |  |  |  |  |  |  |  |  |  |  |  |  |  |  |  |  |  |  |  |  |  |  |  |  |  |  |  |  |  |
| Cyathura carinata | 0 | 0 | 0 | 0 | 0 | 7 | 11 | 6 | 11 | 6 | 0 | 0 | 0 | 0 | 0 | 0 | 0 | 0 | 0 | 0 | 0 | 1 | 24 | 8 | 9 | 0 | 0 | 0 | 0 | 0 |
| Lekanesphaera hookeri | 0 | 0 | 0 | 0 | 0 | 0 | 4 | 3 | 0 | 1 | 0 | 0 | 0 | 0 | 0 | 0 | 0 | 0 | 0 | 0 | 1 | 7 | 27 | 21 | 16 | 0 | 0 | 0 | 0 | 0 |
| Corophium volutator | 1 | 3 | 0 | 2 | 2 | 0 | 0 | 2 | 0 | 1 | 15 | 37 | 38 | 27 | 13 | 3 | 3 | 2 | 1 | 0 | 0 | 0 | 2 | 0 | 0 | 25 | 7 | 19 | 21 | 3 |
| Amphithoe rubricata | 0 | 0 | 0 | 0 | 0 | 0 | 0 | 0 | 0 | 0 | 0 | 0 | 0 | 0 | 0 | 0 | 0 | 0 | 0 | 0 | 0 | 0 | 0 | 0 | 0 | 0 | 0 | 0 | 0 | 0 |
| Tanaidae spp. | 0 | 0 | 0 | 0 | 0 | 0 | 0 | 0 | 0 | 0 | 0 | 0 | 0 | 0 | 0 | 0 | 0 | 0 | 0 | 0 | 0 | 0 | 0 | 0 | 0 | 0 | 0 | 0 | 0 | 0 |
| Idoitea chelipes | 0 | 0 | 0 | 0 | 0 | 0 | 1 | 0 | 1 | 0 | 0 | 0 | 0 | 0 | 0 | 0 | 0 | 0 | 0 | 0 | 0 | 0 | 0 | 0 | 0 | 0 | 0 | 1 | 0 | 0 |
| Gammarus salinus | 0 | 0 | 0 | 0 | 1 | 0 | 0 | 0 | 0 | 1 | 0 | 0 | 0 | 0 | 0 | 0 | 0 | 0 | 0 | 0 | 0 | 0 | 0 | 0 | 0 | 0 | 0 | 0 | 0 | 0 |
| Microdeutopus gryllotalpa | 2 | 0 | 0 | 1 | 0 | 0 | 0 | 0 | 0 | 0 | 0 | 0 | 0 | 0 | 0 | 0 | 0 | 0 | 0 | 0 | 0 | 0 | 0 | 0 | 0 | 0 | 0 | 0 | 0 | 0 |
| Carcinus maenas | 0 | 0 | 0 | 0 | 0 | 0 | 0 | 0 | 0 | 0 | 0 | 0 | 0 | 0 | 0 | 0 | 0 | 2 | 2 | 0 | 0 | 0 | 0 | 0 | 0 | 0 | 0 | 0 | 0 | 0 |
| Tanais dulongii | 0 | 0 | 0 | 0 | 0 | 0 | 0 | 0 | 0 | 0 | 1 | 0 | 0 | 0 | 0 | 0 | 0 | 0 | 0 | 0 | 0 | 0 | 0 | 0 | 0 | 12 | 73 | 51 | 96 | 3 |
| Gammaridae spp. | 0 | 1 | 0 | 0 | 0 | 0 | 0 | 0 | 0 | 0 | 0 | 0 | 0 | 0 | 0 | 0 | 1 | 0 | 1 | 0 | 0 | 0 | 0 | 1 | 1 | 0 | 0 | 0 | 0 | 0 |
| Gammarus duebeni | 0 | 0 | 0 | 0 | 0 | 0 | 0 | 0 | 0 | 0 | 0 | 0 | 0 | 0 | 0 | 0 | 0 | 0 | 0 | 0 | 0 | 0 | 0 | 0 | 0 | 2 | 0 | 0 | 0 | 0 |
| Palaemon adspersus | 0 | 0 | 0 | 0 | 0 | 0 | 0 | 0 | 0 | 0 | 0 | 0 | 0 | 0 | 0 | 0 | 0 | 1 | 1 | 0 | 0 | 0 | 0 | 0 | 0 | 0 | 0 | 0 | 0 | 0 |
| Mysida sp. | 0 | 0 | 0 | 0 | 0 | 0 | 0 | 0 | 0 | 0 | 0 | 0 | 0 | 0 | 0 | 0 | 0 | 1 | 0 | 0 | 0 | 0 | 0 | 0 | 0 | 0 | 0 | 0 | 0 | 0 |
| Erichthonius difformis | 0 | 0 | 0 | 0 | 0 | 0 | 0 | 0 | 0 | 0 | 0 | 0 | 0 | 0 | 0 | 0 | 0 | 0 | 4 | 0 | 0 | 0 | 0 | 0 | 0 | 0 | 0 | 0 | 0 | 0 |
| Chironomida spp. | 4 | 0 | 0 | 0 | 7 | 0 | 0 | 0 | 0 | 0 | 12 | 5 | 2 | 2 | 1 | 0 | 1 | 3 | 3 | 0 | 0 | 0 | 0 | 0 | 0 | 56 | 22 | 42 | 8 | 73 |
| Coleoptera spp. | 0 | 0 | 0 | 0 | 0 | 0 | 0 | 0 | 0 | 0 | 0 | 0 | 0 | 1 | 0 | 0 | 0 | 0 | 0 | 0 | 0 | 0 | 1 | 0 | 1 | 0 | 0 | 0 | 0 | 0 |
| Anurida maritima | 0 | 0 | 0 | 0 | 0 | 0 | 0 | 0 | 0 | 0 | 0 | 0 | 0 | 0 | 0 | 0 | 0 | 2 | 0 | 0 | 48 | 0 | 0 | 2 | 0 | 0 | 0 | 0 | 0 | 0 |
| Limecola balthica | 0 | 1 | 0 | 1 | 1 | 0 | 0 | 0 | 1 | 0 | 3 | 0 | 0 | 2 | 4 | 0 | 2 | 3 | 3 | 1 | 0 | 0 | 0 | 0 | 0 | 6 | 1 | 1 | 1 | 5 |
| Mytilus edulis | 0 | 0 | 0 | 0 | 0 | 0 | 0 | 0 | 0 | 0 | 0 | 0 | 0 | 1 | 0 | 2 | 1 | 1 | 0 | 0 | 0 | 1 | 2 | 0 | 2 | 0 | 0 | 0 | 0 | 0 |
| Spisula subtruncata | 0 | 0 | 0 | 0 | 0 | 0 | 0 | 0 | 0 | 0 | 0 | 0 | 0 | 0 | 0 | 0 | 0 | 0 | 0 | 0 | 0 | 0 | 0 | 0 | 0 | 0 | 0 | 0 | 0 | 0 |
| Polititapes rhomboides | 0 | 0 | 0 | 0 | 0 | 0 | 0 | 0 | 0 | 0 | 0 | 0 | 0 | 0 | 0 | 0 | 3 | 0 | 0 | 0 | 0 | 0 | 0 | 0 | 0 | 0 | 0 | 0 | 0 | 0 |
| Parvicardium scabrum | 0 | 0 | 0 | 0 | 0 | 0 | 0 | 0 | 0 | 0 | 0 | 0 | 0 | 0 | 0 | 0 | 0 | 0 | 0 | 0 | 0 | 0 | 0 | 0 | 0 | 0 | 0 | 0 | 0 | 0 |
| Cerastoderma edule | 1 | 3 | 0 | 0 | 0 | 0 | 0 | 0 | 0 | 0 | 0 | 0 | 0 | 0 | 0 | 4 | 3 | 1 | 4 | 4 | 0 | 0 | 0 | 0 | 0 | 0 | 0 | 0 | 0 | 0 |
| Cerastoderma glaucum | 0 | 0 | 0 | 0 | 0 | 0 | 0 | 0 | 1 | 0 | 4 | 0 | 1 | 1 | 12 | 0 | 0 | 0 | 0 | 0 | 1 | 0 | 7 | 14 | 14 | 6 | 0 | 4 | 6 | 4 |
| Scrobicularia plana | 0 | 0 | 0 | 0 | 0 | 0 | 0 | 0 | 0 | 0 | 0 | 0 | 0 | 0 | 0 | 0 | 0 | 0 | 0 | 0 | 0 | 0 | 0 | 0 | 0 | 0 | 0 | 0 | 0 | 0 |
| Abra tenius | 0 | 0 | 0 | 0 | 0 | 0 | 0 | 0 | 0 | 0 | 0 | 0 | 0 | 0 | 0 | 2 | 1 | 3 | 0 | 0 | 0 | 0 | 0 | 0 | 0 | 0 | 0 | 0 | 0 | 0 |
| Crassostrea gigas | 0 | 0 | 0 | 0 | 0 | 0 | 0 | 0 | 0 | 0 | 0 | 0 | 0 | 0 | 0 | 0 | 1 | 0 | 0 | 0 | 0 | 0 | 0 | 0 | 0 | 0 | 0 | 0 | 0 | 0 |
| Modiolula phaseolina | 0 | 0 | 0 | 0 | 0 | 0 | 0 | 0 | 0 | 0 | 0 | 0 | 0 | 0 | 0 | 2 | 6 | 6 | 19 | 0 | 0 | 0 | 0 | 0 | 0 | 0 | 0 | 0 | 0 | 0 |
| Mya arenaria | 3 | 5 | 1 | 3 | 0 | 4 | 2 | 4 | 5 | 2 | 0 | 0 | 0 | 1 | 5 | 2 | 5 | 4 | 0 | 0 | 1 | 1 | 2 | 2 | 5 | 1 | 0 | 1 | 1 | 1 |
| Parvicardium ovale | 0 | 0 | 0 | 0 | 0 | 0 | 0 | 0 | 0 | 0 | 0 | 0 | 0 | 0 | 0 | 2 | 1 | 3 | 0 | 0 | 0 | 0 | 0 | 0 | 0 | 0 | 0 | 0 | 0 | 0 |
| Hydrobia spp. | 31 | 28 | 22 | 59 | 23 | 245 | 206 | 936 | 332 | 267 | 1 | 1 | 1 | 9 | 20 | 144 | 93 | 77 | 417 | 111 | 504 | 584 | 285 | 336 | 1325 | 8 | 2 | 1 | 8 | 1 |
| Theodoxus fluviatilis | 0 | 0 | 0 | 0 | 0 | 0 | 0 | 1 | 0 | 0 | 0 | 0 | 0 | 0 | 0 | 0 | 0 | 0 | 0 | 0 | 2 | 3 | 2 | 2 | 1 | 0 | 0 | 0 | 0 | 0 |
| Bittium reticulatum | 7 | 15 | 9 | 16 | 12 | 0 | 0 | 0 | 0 | 0 | 0 | 0 | 0 | 0 | 0 | 10 | 5 | 0 | 9 | 0 | 0 | 0 | 0 | 0 | 0 | 0 | 0 | 0 | 0 | 0 |
| Nassarius reticulatus | 0 | 0 | 0 | 0 | 0 | 0 | 0 | 0 | 0 | 0 | 0 | 0 | 0 | 0 | 0 | 0 | 0 | 0 | 0 | 0 | 0 | 0 | 0 | 0 | 0 | 0 | 0 | 0 | 0 | 0 |
| Radix peregra | 0 | 0 | 0 | 0 | 0 | 0 | 0 | 0 | 0 | 0 | 0 | 0 | 0 | 0 | 0 | 0 | 0 | 0 | 0 | 0 | 0 | 0 | 0 | 0 | 0 | 0 | 0 | 0 | 0 | 0 |
| Littorina spp. | 1 | 0 | 0 | 4 | 0 | 0 | 0 | 0 | 0 | 0 | 0 | 0 | 0 | 0 | 0 | 0 | 3 | 0 | 0 | 0 | 4 | 0 | 3 | 7 | 5 | 0 | 0 | 0 | 0 | 0 |
| Pusillina sarsi | 0 | 0 | 0 | 0 | 0 | 0 | 0 | 0 | 0 | 0 | 0 | 0 | 0 | 0 | 0 | 1 | 0 | 31 | 21 | 0 | 0 | 0 | 0 | 0 | 0 | 0 | 0 | 0 | 0 | 0 |
| Retusa umbillicata | 0 | 0 | 0 | 0 | 0 | 0 | 0 | 0 | 0 | 0 | 0 | 0 | 0 | 0 | 0 | 1 | 3 | 2 | 0 | 2 | 0 | 0 | 0 | 0 | 0 | 0 | 0 | 0 | 0 | 0 |
| Retusa truncatula | 0 | 0 | 0 | 0 | 0 | 0 | 0 | 0 | 0 | 0 | 0 | 0 | 0 | 0 | 0 | 0 | 0 | 0 | 4 | 0 | 0 | 0 | 0 | 0 | 0 | 0 | 0 | 0 | 0 | 0 |
| Majidae sp | 0 | 0 | 0 | 0 | 0 | 0 | 0 | 0 | 0 | 0 | 0 | 0 | 0 | 0 | 0 | 0 | 0 | 0 | 1 | 0 | 0 | 0 | 0 | 0 | 0 | 0 | 0 | 0 | 0 | 0 |
| Elysia viridis | 0 | 0 | 0 | 0 | 0 | 0 | 0 | 0 | 0 | 0 | 0 | 0 | 0 | 0 | 0 | 0 | 0 | 0 | 0 | 0 | 0 | 0 | 0 | 0 | 0 | 0 | 0 | 0 | 0 | 0 |
| Lepidochiton cinereus | 0 | 0 | 0 | 0 | 0 | 0 | 0 | 0 | 0 | 0 | 0 | 0 | 0 | 0 | 0 | 1 | 3 | 0 | 0 | 0 | 0 | 0 | 0 | 0 | 0 | 0 | 0 | 0 | 0 | 0 |
| **Nematoda** |  |  |  |  |  |  |  |  |  |  |  |  |  |  |  |  |  |  |  |  |  |  |  |  |  |  |  |  |  |  |
| Nematoda spp. | 0 | 3 | 0 | 7 | 6 | 0 | 0 | 1 | 0 | 0 | 1 | 0 | 0 | 0 | 1 | 0 | 0 | 3 | 3 | 0 | 0 | 0 | 2 | 0 | 1 | 2 | 0 | 0 | 0 | 1 |
|  |  |  |  |  |  |  |  |  |  |  |  |  |  |  |  |  |  |  |  |  |  |  |  |  |  |  |  |  |  |  |

Supplementary table 2. Abundance of focal species for genetic diversity sampling at all sample sites and time (H – high abundance, M – medium abundance, L – low abundance, A – absent), abundance is based on subjective observed frequency in samples. Asterisks indicate samples not included in the genetic analyses due to low abundance.

| **Site** | Sample  time | *H. diversicolor* | *P. elegans* | *M. balthica* | *M. arenaria* | *M. edulis* | *C. volutator* |
| --- | --- | --- | --- | --- | --- | --- | --- |
| List | August 2018 | H | H | H | H | H | H |
| Saltö | August 2018 | H | H | L | M | H | A* |
|  | November 2018 | L* | H | L | H | H | A* |
|  | April 2019 | H | H | M | H | H | H |
|  | August 2019 | H | H | L* | H | H | H |
| Herslev | August 2018 | H | H | L | M | H | H |
|  | November 2018 | H | H | M | H | H | H |
|  | April 2019 | H | H | M | H | H | H |
|  | August 2019 | H | H | L | M | H | H |
| Gollwitz | August 2018 | H | H | H | H | H | H |
| Öland | August 2018 | H | A* | H | M | H | H |
|  | November 2018 | H | A* | H | H | H | H |
|  | April 2019 | H | L* | H | H | H | H |
|  | August 2019 | H | L* | H | H | H | H |
| Tvärminne | August 2018 | H | A* | H | L* | M | H |
| Pori | August 2018 | M | A* | M | L* | L | A |

Supplementary table 3: Environmental factors explaining Shannon diversity and nucleotide diversity for each dataset according to general linear mixed modelling. P-values in bold are significant at p ≤ 0.05, underlined p-values are significant at p ≤ 0.1. Note that intercept refers to random effects.

| ***Hediste diversicolor* dataset** | | | | | |  |  | | | | | | | | |
| --- | --- | --- | --- | --- | --- | --- | --- | --- | --- | --- | --- | --- | --- | --- | --- |
| **Shannon diversity** | | | | | |  | **Nucleotide diversity** | | | | | | | | |
| Random effects (Poisson) | | | | | |  | Random effects (Normal) | | | | | | | | |
| **Coefficient** | Estimate | SE | df | *t* | p |  | **Coefficient** | Estimate | SE | df | | *t* | p | |  |
| Intercept | -0.448 | 1.421 | 7 | -0.316 | 0.762 |  | Intercept | -5.368 | 1.172 | 7 | -4.581 | | | **0.003** |  |
| Salinity | 0.001 | 0.003 | 7 | -0.016 | 0.988 |  | Salinity | 0.006 | 0.024 | 7 | 0.245 | | | 0.814 |  |
| Temperature | 0.004 | 0.023 | 7 | 0.148 | 0.886 |  | Temperature | -0.037 | 0.017 | 7 | -2.197 | | | 0.064 |  |
| C/N ratio | 0.046 | 0.057 | 7 | -0.806 | 0.450 |  | C/N ratio | 0.048 | 0.049 | 7 | 0.977 | | | 0.361 |  |
| Organic matter | -0.040 | 0.452 | 7 | -0.087 | 0.933 |  | Organic matter | -0.354 | 0.309 | 7 | -1.144 | | | 0.290 |  |
| Porosity | 1.825 | 2.756 | 7 | -0.662 | 0.529 |  | Porosity | -0.398 | 1.997 | 7 | -0.199 | | | 0.848 |  |
| Mean grain size | -0.048 | 0.378 | 7 | -0.013 | 0.902 |  | Mean grain size | 0.110 | 0.332 | 7 | 0.330 | | | 0.751 |  |
| Sorting | 0.284 | 0.427 | 7 | 0.666 | 0.527 |  | Sorting | -0.104 | 0.341 | 7 | -0.306 | | | 0.769 |  |
|  |  |  |  |  |  |  |  |  |  |  | |  |  | |  |

| ***Pygospio elegans* dataset** | | | | | |  |  | | | | | | |
| --- | --- | --- | --- | --- | --- | --- | --- | --- | --- | --- | --- | --- | --- |
| **Shannon diversity** | | | | | |  | **Nucleotide diversity** | | | | | | |
| Random effects (Poisson) | | | | | |  | Random effects (Normal) | | | | | | |
| **Coefficient** | Estimate | SE | df | *t* | p |  | **Coefficient** | Estimate | SE | df | *t* | p |  |
| Intercept | -2.269 | 4.609 | 1 | -0.492 | 0.709 |  | Intercept | -6.066 | 0.583 | 1 | -10.405 | 0.061 |  |
| Salinity | 0.068 | 0.179 | 1 | 0.381 | 0.769 |  | Salinity | 0.012 | 0.025 | 1 | 0.476 | 0.717 |  |
| Temperature | 0.003 | 0.069 | 1 | 0.040 | 0.975 |  | Temperature | -0.015 | 0.010 | 1 | -1.488 | 0.377 |  |
| C/N ratio | -0.032 | 0.240 | 1 | -0.131 | 0.917 |  | C/N ratio | -0.043 | 0.033 | 1 | -1.304 | 0.416 |  |
| Organic matter | 2.565 | 6.117 | 1 | 0.419 | 0.747 |  | Organic matter | 2.134 | 0.765 | 1 | 2.789 | 0.219 |  |
| Porosity | -1.956 | 12.509 | 1 | -0.156 | 0.901 |  | Porosity | -3.495 | 1.600 | 1 | -2.184 | 0.273 |  |
| Mean grain size | -0.106 | 0.944 | 1 | -0.112 | 0.929 |  | Mean grain size | -0.100 | 0.146 | 1 | -0.687 | 0.617 |  |
| Sorting | -0.076 | 1.345 | 1 | -0.056 | 0.964 |  | Sorting | 0.227 | 0.192 | 1 | 1.180 | 0.448 |  |
|  |  |  |  |  |  |  |  |  |  |  |  |  |  |

| ***Macoma balthica* dataset** | | | | | |  |  | | | | | | | |
| --- | --- | --- | --- | --- | --- | --- | --- | --- | --- | --- | --- | --- | --- | --- |
| **Shannon diversity**  Random effects (Poisson) | | | | | |  | **Nucleotide diversity**  Random effects (Normal) | | | | | | | |
| **Coefficient** | Estimate | SE | df | *t* | p |  | **Coefficient** | Estimate | SE | df | | *t* | p |  |
| Intercept | 0.228 | 0.920 | 7 | 0.248 | 0.811 |  | Intercept | -7.463 | 0.856 | 7 | -8.721 | | **5.2e-05** |  |
| Salinity | 0.028 | 0.016 | 7 | 1.755 | 0.123 |  | Salinity | 0.021 | 0.014 | 7 | 1.459 | | 0.188 |  |
| Temperature | -0.017 | 0.016 | 7 | -1.049 | 0.329 |  | Temperature | 0.032 | 0.014 | 7 | 2.391 | | **0.048** |  |
| C/N ratio | 0.097 | 0.039 | 7 | 2.516 | **0.040** |  | C/N ratio | -0.056 | 0.030 | 7 | -1.858 | | 0.105 |  |
| Organic matter | 0.262 | 0.317 | 7 | 0.827 | 0.435 |  | Organic matter | -0.564 | 0.285 | 7 | -1.978 | | 0.089 |  |
| Porosity | -1.619 | 1.993 | 7 | -0.816 | 0.443 |  | Porosity | 4.308 | 1.897 | 7 | 2.271 | | 0.057 |  |
| Mean grain size | 0.226 | 0.241 | 7 | 0.938 | 0.379 |  | Mean grain size | 0.139 | 0.190 | 7 | 0.733 | | 0.487 |  |
| Sorting | -0.638 | 0.297 | 7 | -2.148 | 0.069 |  | Sorting | 0.160 | 0.234 | 7 | 0.685 | | 0.516 |  |
|  |  |  |  |  |  |  |  |  |  |  |  | |  |  |

| ***Mya arenaria* dataset** | | | | | |  |  | | | | | | | |
| --- | --- | --- | --- | --- | --- | --- | --- | --- | --- | --- | --- | --- | --- | --- |
| **Shannon diversity**  Random effects (Poisson) | | | | | |  | **Nucleotide diversity**  Random effects (Normal) | | | | | | | |
|  | | | | | |  |  | | | | | | | |
| **Coefficient** | Estimate | SE | df | *t* | p |  | **Coefficient** | Estimate | | SE | df | *t* | p |  |
| Intercept | -0.325 | 1.516 | 6 | -0.215 | 0.837 |  | Intercept | -5.986 | 0.601 | | 6 | -9.964 | **5.9e-05** |  |
| Salinity | 0.011 | 0.027 | 6 | 0.394 | 0.708 |  | Salinity | 0.000 | 0.010 | | 6 | 0.040 | 0.969 |  |
| Temperature | -0.003 | 0.026 | 6 | -0.112 | 0.914 |  | Temperature | -0.007 | 0.010 | | 6 | -0.652 | 0.539 |  |
| C/N ratio | 0.055 | 0.062 | 6 | 0.888 | 0.409 |  | C/N ratio | 0.017 | 0.026 | | 6 | 0.653 | 0.538 |  |
| Organic matter | -0.272 | 0.657 | 6 | -0.429 | 0.683 |  | Organic matter | -0.279 | 0.247 | | 6 | -1.128 | 0.303 |  |
| Porosity | 0.741 | 3.556 | 6 | 0.208 | 0.842 |  | Porosity | 3.302 | 1.423 | | 6 | 2.320 | 0.060 |  |
| Mean grain size | 0.115 | 0.410 | 6 | 0.280 | 0.789 |  | Mean grain size | -0.272 | 0.154 | | 6 | -1.764 | 0.128 |  |
| Sorting | 0.211 | 0.389 | 6 | 0.543 | 0.607 |  | Sorting | -0.037 | 0.140 | | 6 | -0.267 | 0.798 |  |
|  |  |  |  |  |  |  |  |  |  | |  |  |  |  |

| ***Mytilus edulis* dataset** | | | | | |  |  | | | | | | |
| --- | --- | --- | --- | --- | --- | --- | --- | --- | --- | --- | --- | --- | --- |
| **Shannon diversity**  Random effects (Poisson) | | | | | |  | **Nucleotide diversity**  Random effects (Normal) | | | | | | |
|  | | | | | |  |  | | | | | | |
| **Coefficient** | Estimate | SE | df | *t* | p |  | **Coefficient** | Estimate | SE | df | *t* | p |  |
| Intercept | -0.526 | 1.157 | 7 | -0.455 | 0.663 |  | Intercept | -8.148 | 1.390 | 7 | -5.861 | **0.001** |  |
| Salinity | 0.009 | 0.021 | 7 | 0.440 | 0.673 |  | Salinity | 0.090 | 0.020 | 7 | 4.601 | **0.003** |  |
| Temperature | -0.003 | 0.021 | 7 | -0.130 | 0.900 |  | Temperature | 0.039 | 0.021 | 7 | 1.915 | 0.097 |  |
| C/N ratio | 0.054 | 0.049 | 7 | 1.116 | 0.301 |  | C/N ratio | -0.096 | 0.041 | 7 | -2.371 | **0.050** |  |
| Organic matter | -0.326 | 0.512 | 7 | -0.635 | 0.545 |  | Organic matter | -0.255 | 0.597 | 7 | -0.427 | 0.682 |  |
| Porosity | 1.420 | 2.539 | 7 | 0.559 | 0.593 |  | Porosity | 3.071 | 3.298 | 7 | 0.931 | 0.383 |  |
| Mean grain size | 0.088 | 0.312 | 7 | 0.283 | 0.786 |  | Mean grain size | 0.595 | 0.277 | 7 | 2.148 | 0.069 |  |
| Sorting | 0.291 | 0.306 | 7 | 0.952 | 0.373 |  | Sorting | -0.782 | 0.275 | 7 | -2.845 | **0.025** |  |
|  |  |  |  |  |  |  |  |  |  |  |  |  |  |

| ***Corophium volutator* dataset** | | | | | |  |  | | | | | | |
| --- | --- | --- | --- | --- | --- | --- | --- | --- | --- | --- | --- | --- | --- |
| **Shannon diversity**  Random effects (Poisson) | | | | | |  | **Nucleotide diversity**  Random effects (Normal) | | | | | | |
|  | | | | | |  |  | | | | | | |
| **Coefficient** | Estimate | SE | df | *t* | p |  | **Coefficient** | Estimate | SE | df | *t* | p |  |
| Intercept | 0.418 | 0.827 | 3 | 0.505 | 0.648 |  | Intercept | -6.792 | 0.791 | 3 | -8.592 | **0.003** |  |
| Salinity | 0.050 | 0.027 | 3 | 1.839 | 0.163 |  | Salinity | 0.004 | 0.028 | 3 | 0.150 | 0.890 |  |
| Temperature | -0.009 | 0.015 | 3 | -0.587 | 0.599 |  | Temperature | 0.026 | 0.013 | 3 | 1.976 | 0.143 |  |
| C/N ratio | 0.093 | 0.039 | 3 | 2.378 | 0.099 |  | C/N ratio | -0.072 | 0.024 | 3 | -2.939 | 0.061 |  |
| Organic matter | 0.698 | 0.683 | 3 | 1.023 | 0.382 |  | Organic matter | -0.240 | 0.687 | 3 | -0.349 | 0.750 |  |
| Porosity | -1.934 | 2.259 | 3 | -0.056 | 0.455 |  | Porosity | 2.508 | 2.369 | 3 | 1.059 | 0.367 |  |
| Mean grain size | 0.197 | 0.207 | 3 | 0.954 | 0.411 |  | Mean grain size | -0.069 | 0.195 | 3 | 0.352 | 0.748 |  |
| Sorting | -1.730 | 1.099 | 3 | -1.574 | 0.214 |  | Sorting | 0.492 | 1.088 | 3 | 0.453 | 0.682 |  |
|  |  |  |  |  |  |  |  |  |  |  |  |  |  |

Supplementary table 4. GenBank assembly accession number for all species included in probe design, ordered by taxonomic group.

| Species group | Species | GenBank assembly accession number |
| --- | --- | --- |
| Annelida | *Amynthas corticis* | GCA_900184025.1 |
|  | *Capitella teleta* | GCA_000328365.1 |
|  | *Eisenia fetida* | GCA_003999395.1 |
|  | *Hydroides elegans* | GCA_001703475.1 |
|  | *Lamellibranchia luymesi* | GCA_009193005.1 |
| Bivalvia | *Dreissena rostriformis* | GCA_007657795.1 |
|  | *Lutraria rhynchaena* | GCA_008271625.1 |
|  | *Modiolus philippinarum* | GCA_002080025.1 |
|  | *Mytilus galloprovincialis* | GCA_001676915.1 |
|  | *Sinonovacula constricta* | GCA_009762815.1 |
| Gastropoda | *Babylonia areolata* | GCA_011634625.1 |
|  | *Cumia reticulata* | GCA_900004695.1 |
|  | *Lanistes nyassanus* | GCA_004794575.1 |
|  | *Marisa cornuarietis* | GCA_004794655.1 |
|  | *Pomacea maculata* | GCA_004794325.1 |
| Crustacea | *Hyalella azteca* | GCA_000764305.2 |
|  | *Ligia exotica* | GCA_002091915.1 |
|  | *Parhyale hawaiensis* | GCA_001587735.2 |
|  | *Platorchestia hallaensis* | GCA_014220935.1 |
|  | *Trinorchestia longiramus* | GCA_006783055.1 |

Supplementary Figure 1. Flow diagram of bioinformatic procedure and analyses.

Reads length, adapter and quality trimmed

Reference loci: reads assembled to contigs and aligned to probe set

Matrix of UCE loci

Fasta-file with UCE loci for each sample

Reads mapped to Fasta-file of UCE loci

SNPs calculated for each UCE locus
